# Phenotypic, PCR, and whole-genome sequencing characterization of carbapenem-resistant *Acinetobacter baumannii* in Makkah and Riyadh, Saudi Arabia: AST discordance and local genomic micro-clusters

**DOI:** 10.3389/fmicb.2026.1845444

**Published:** 2026-06-22

**Authors:** Azzam A. Alquait, Basel M. Alnafjan, Mohammed H. Abutarboush, Shouq F. Algannam, Mohammed A. Alolayan, Abdulaziz A. Huraysi, Amjad N. Alotaibi, Abeer H. Alomair, Abdulmalek T. Algarni, Basem J. Almutiri, Fatima F. Alghannam, Hatim Almutairi, Sarah S. Alotaibi, Amal M. Alsubhi, Fahhad D. Alsahli, Maryam K. Alshammari, Seham S. Alharthi, Fahad M. Alhoshani, Fayez S. Bahwerth, Turki M. Dawoud, Bandar K. Sendy, Essam A. Tawfik, Ahmed A. Al-Qahtani, Musaad A. Altammami, Ahmad M. Aldossary, Essam J. Alyamani

**Affiliations:** 1 Health Sector, King Abdulaziz City for Science and Technology, Wellness and Preventive Medicine Institute, Riyadh, Saudi Arabia; 2 Health Sector, King Abdulaziz City for Science and Technology, Applied Genomics Technologies Institute, Riyadh, Saudi Arabia; 3 Health Sector, King Abdulaziz City for Science and Technology, Advanced Diagnostics and Therapeutics Institute, Riyadh, Saudi Arabia; 4 Department of Infection and Immunity, Research Centre, King Faisal Specialist Hospital and Research Centre, Riyadh, Saudi Arabia; 5 Department of Genome and Data, National Livestock and Fisheries Development Program, Ministry of Environment, Water and Agriculture, Riyadh, Saudi Arabia; 6 Health Sector, King Abdulaziz City for Science and Technology, Bioengineering Institute, Riyadh, Saudi Arabia; 7 College of Applied Medical Sciences, Inaya Medical College, Riyadh, Saudi Arabia; 8 Microbiology Department, Hera General Hospital, Ministry of Health, Makkah, Saudi Arabia; 9 Department of Botany and Microbiology, College of Science, King Saud University, Riyadh, Saudi Arabia

**Keywords:** *Acinetobacter baumannii*, carbapenem resistance, infection prevention and control, outbreak micro-clusters, whole-genome sequencing

## Abstract

**Background:**

Carbapenem-resistant *Acinetobacter baumannii* (CRAB) is a World Health Organization (WHO) critical-priority pathogen, yet hospitals need practical workflows that link rapid rule-in testing, definitive minimum inhibitory concentration (MIC) confirmation, and actionable genomic thresholds for outbreak response. Integrated phenotype–polymerase chain reaction (PCR)–whole-genome sequencing (WGS) characterization studies with decision-ready performance metrics remain limited for settings in Saudi Arabia.

**Methods:**

We analyzed 71 non-duplicate multidrug-resistant (MDR) *A. baumannii* clinical isolates from two cities in Saudi Arabia (Riyadh, *n* = 36; Makkah, *n* = 35), collected from 2024 to 2025. We compared VITEK II antimicrobial susceptibility testing (AST) to Clinical and Laboratory Standards Institute (CLSI) broth microdilution (BMD) MICs for imipenem (IMI), meropenem (MEM), ceftazidime (CAZ), and ciprofloxacin (CIP); evaluated targeted PCR for *blaOXA-23* and *blaNDM* (with *blaOXA-51-like* confirmation); and performed whole-genome sequencing (WGS; *n* = 59 passing quality control [QC]) for recombination-filtered core-single nucleotide polymorphism (SNP) phylogeny, multilocus sequence typing (MLST), capsular locus (KL)/lipooligosaccharide outer-core locus (OCL) typing, resistome profiling, and SNP-threshold micro-cluster definition.

**Results:**

By BMD, carbapenem resistance was essentially universal (imipenem [IMI] 100% in both cities; meropenem [MEM] 100% in Makkah and 97.2% in Riyadh). VITEK II software showed high categorical agreement with BMD (94–100% across drug city strata) but non-zero very major error (VME) rates for carbapenems (up to 5.9%), supporting reflex BMD confirmation for decision-changing susceptible calls. The composite PCR marker (*blaOXA-23* or *blaNDM*) achieved high sensitivity for imipenem resistance (100% in Makkah; 91.7% in Riyadh). WGS revealed global clone 2 (GC2)/sequence type 2 (ST2) predominance with interleaved Riyadh–Makkah clades (regional circulation) but predominantly single-city ≤10-SNP micro-clusters (0 mixed-city clusters; largest: 22 isolates in Makkah; largest Riyadh cluster: 5 isolates), indicating putative recent local transmission or shared-source signals.

**Conclusion:**

A combined same-day PCR rule-in, reference MIC confirmation, and WGS micro-cluster surveillance strategy operationalizes rapid CRAB triage and outbreak triggering in hospitals in Saudi Arabia, enabling proactive infection-prevention containment.

## Background

*Acinetobacter baumannii* (*A. baumannii*) has emerged as one of the most problematic nosocomial pathogens worldwide, driven by an unusual capacity to survive desiccation, persist in hospital environments, and rapidly accumulate antimicrobial resistance (AMR) determinants. Reflecting this threat, the World Health Organization (2024) Bacterial Priority Pathogens List categorized carbapenem-resistant *A. baumannii* (CRAB) in the critical-priority tier, where urgent action is required to guide research and development, and public-health interventions (World Health Organization, 2024). Clinically, CRAB is associated with ventilator-associated pneumonia, bloodstream and wound infections in high-acuity settings, where therapeutic options are limited, and outcomes are poor; contemporary syntheses estimate mortality >20% overall and frequently >40% for bloodstream infection, with substantial regional variability linked to resistance epidemiology, access to active agents, and infection-prevention capacity (Karakonstantis et al., 2020; Castanheira et al., 2023).

The global AMR architecture of CRAB is anchored in the spread of OXA-type carbapenemases, especially *blaOXA-23*, which are frequently carried by highly adapted international clone 2 ([IC2]/global clone 2 [GC2]) lineages that dominate hospital outbreaks across continents. Mechanistic and genomic reviews consistently identify *blaOXA-23* as the most prevalent acquired carbapenemase worldwide, with additional contributions from *blaNDM* metallo-*β*-lactamases in some settings (Hamidian and Hall, 2018; Martins-Gonçalves et al., 2024). Beyond carbapenemases, resistance is layered by RND efflux pumps (*AdeABC*, *AdeIJK*, and *AdeFGH*), 16S rRNA methyltransferases (e.g., *armA*), aminoglycoside-modifying enzymes, macrolide efflux (*msrE/mphE*), and tetracycline regulators (*tet(B)/tetR*), yielding the multidrug-resistant (MDR)/extensively multidrug-resistant (XDR) phenotypes commonly seen in intensive care units (ICUs) (Coyne et al., 2011; Shi, 2024). These molecular features help explain the frequent discordance between automated antimicrobial susceptibility testing (AST) and clinical efficacy, the need for reference minimum inhibitory concentration (MIC) testing, and the narrow reliance on agents such as polymyxins, sulbactam-based combinations, and Cefiderocol in the most severely ill patients (Shi, 2024).

Regional epidemiology in the Middle East and Gulf mirrors the global picture, but with exceptionally high carbapenem resistance rates and a strong presence of *blaOXA-23* producers. A seminal multicountry study across the Gulf Cooperation Council (GCC) states reported the dominance of *blaOXA-23-type* CRAB, underscoring cross-border circulation of high-risk clones in regional hospital networks (Zowawi et al., 2015). Subsequent reviews and country-level reports indicate that CRAB prevalence approaches or exceeds 80–90% in several Middle Eastern settings, highlighting the clinical and economic burden on intensive care medicine (Al-Rashed et al., 2021).

Within Saudi Arabia, multiple studies and syntheses document a sustained, high burden of MDR/XDR *A. baumannii*. The comprehensive review by Ibrahim collated national data and emphasized the convergence of risk factors (critical illness, device use, antimicrobial pressure) with mechanisms of carbapenem resistance centered on OXA-type enzymes (Ibrahim, 2019). More recent overviews of surveillance in Saudi Arabia echo these trends and call for coordinated infection prevention and stewardship, given the repeated detection of CRAB in ICUs and the challenges of empiric therapy (Alharbi et al., 2025; Thabit et al., 2025). Environmental work from the Western region further demonstrates that CRAB can persist in hospital environments, with whole-genome sequencing (WGS) revealing relatedness between clinical and environmental isolates, evidence that reservoir control is integral to prevention (Yasir et al., 2022). Together, these studies suggest that hospitals in Saudi Arabia face an endemic CRAB, characterized by the same global high-risk lineages but modulated by local ecology and care pathways.

Against this backdrop, molecular epidemiology in Saudi Arabia has contributed foundational insights. Notably, Alyamani et al. (2015) characterized extended-spectrum β-lactamases (ESBLs) in *A. baumannii* from clinical isolates in Saudi Arabia, documenting diverse β-lactamase content and underlining the genetic heterogeneity that can coexist with carbapenem resistance in local collections. Such national studies complement regional GCC analyses and illustrate how genotypic diversity intersects with phenotypic multidrug resistance, reinforcing the need for genomics-informed surveillance (Zowawi et al., 2015; Alharbi et al., 2025). The clinical burden in Saudi Arabia is multidimensional: high CRAB prevalence increases intensive care unit (ICU) length of stay, demands combination or last-line therapy, and is associated with higher mortality relative to carbapenem susceptible infections patterns consistent with global observations (Karakonstantis et al., 2020; Castanheira et al., 2023). As in other settings, inter-facility patient movement and shared care pathways facilitate the dissemination of lineage across cities and hospitals; without genomic tracking, cryptic transmission may go unrecognized until large clusters are established. In response, many centers are pairing routine AST and rapid carbapenemase polymerase chain reaction (PCR) with WGS to delineate transmission, distinguish between reintroduction and persistence, and anchor infection prevention to concrete genomic evidence, an integrated approach repeatedly endorsed in reviews (Peleg et al., 2008; Hamidian and Hall, 2018; Castanheira et al., 2023).

Hospitals in Saudi Arabia lack a validated, end-to-end workflow that quantitatively links routine automated AST, reference MIC testing, focused same-day carbapenemase PCR, and an actionable WGS micro-cluster definition to drive same-day triage and objective outbreak triggers. Without this bridge, hospitals cannot reliably distinguish local transmission that requires immediate containment from the background regional circulation of high-risk clones.

Here, we address this gap by integrating broth microdilution (BMD), VITEK II AST, targeted *blaOXA-23*/*blaNDM* PCR, and WGS across two highly dynamic cities in Saudi Arabia, Riyadh, the Capital, and Makkah, the holiest city in Islam, quantifying diagnostic agreement and error modes, defining a decision-ready ≤10-single nucleotide polymorphism (SNP) micro-cluster threshold, and translating these outputs into a practical infection-prevention and control response framework.

## Methods

### Study setting, isolates, and inclusion criteria

We conducted a cross-sectional laboratory study of 71 MDR *A. baumannii* clinical isolates collected in Saudi Arabia from two sites: Riyadh (*n* = 36) and Makkah (*n* = 35) over 1 year from 2024 to 2025. One isolate per patient was included to ensure non-duplicate inclusion. Isolates were obtained from routine hospital diagnostic specimens, including sputum, wound swabs, tracheal aspirates, bronchial washes, blood, tissue, drains, and urine. De-identification was applied before analysis in accordance with the institutional ethics framework. Full anonymized isolate metadata, including isolate code, city, specimen/source category, BMD phenotype, PCR result, WGS quality control (QC) status, and cluster identity (ID), are provided in Supplementary Table S0. After routine clinical isolation and identification in the hospital microbiology labs, single colonies were subcultured onto standard laboratory media and stored at −80 °C in cryopreservation vials before being cultured on Cation-Adjusted Mueller–Hinton Broth (CAMHB). A laboratory-quality control (QC) strain (ATCC 19606) was run in parallel with susceptibility testing, in accordance with Clinical and Laboratory Standards Institute (CLSI) guidance. QC MICs fell within CLSI M100 ranges on all test days.

### Species identification and routine antimicrobial susceptibility testing (VITEK II)

Species identification and automated AST were performed using the AST-N291 test card and the bioMérieux VITEK II (version 8.01) software according to the manufacturer’s instructions for Gram-negative panels. The antibiotic panel included, at a minimum, imipenem (IMI), meropenem (MEM), ceftazidime (CAZ), cefepime, gentamicin, ciprofloxacin (CIP), tigecycline, and trimethoprim/sulfamethoxazole. VITEK II categorical outputs (S, I, and R) were exported for each isolate and summarized by city (Makkah vs. Riyadh). The susceptibility data were visualized as stacked S/I/R bars, enabling direct comparison of VITEK results with the reference method, BMD. The difference in resistance proportion (*Δ*%R) was calculated as the BMD minus the VITEK value, providing a side-by-side comparison of the methods’ performance.

#### Reference broth microdilution (BMD) MIC testing and CLSI interpretation

The BMD was performed to determine MICs for the 71 *A. baumannii* isolates (Riyadh *n* = 36; Makkah *n* = 35) using cation-adjusted Mueller–Hinton broth (CAMHB) and CLSI M100 (2024) guidance (Clinical and Laboratory Standards Institute, 2024). Antibiotic stock solutions of ciprofloxacin (CIP), ceftazidime (CAZ), meropenem (MEM), and imipenem (IMI) were prepared according to the manufacturers’ instructions and diluted two-fold in CAMHB to yield final test ranges of 1,024–0.5 μg/mL. Two-fold dilutions (50 μL) were dispensed into sterile, flat-bottom 96-well microtiter plates, with a final well volume of 100 μL after inoculation. Inocula were prepared from fresh overnight growth, adjusted to 0.5 McFarland, and diluted in CAMHB to achieve a final well concentration of approximately 5 × 10^5^ CFU/mL. Plates were incubated at 35 ± 2 °C for 16–20 h in ambient air without shaking. MICs were read as the lowest concentration with no visible growth; when needed, Optical Density at 600 nm, OD600 readings were used only to confirm visual endpoints (PowerWave XS2 microplate reader, BioTek Instruments, Winooski, VT, United States). *A. baumannii* ATCC 19606 was included as the quality-control strain, and growth and sterility controls were included on each plate. MICs were interpreted using CLSI M100 (2024) breakpoints, and city-level medians, interquartile ranges (IQRs), ranges, and categorical tallies (S/I/R) were reported in Supplementary Table S1; raw isolate-level MIC values are provided in Supplementary Tables S3, S4.

### PCR detection of Carbapenemase genes

To rapidly characterize carbapenemase genotypes, we performed PCR targeting the *blaOXA-23*, *blaNDM*, and *blaOXA-51-like* genes. For each isolate, DNA was prepared using at least one of three extraction methods available in the dataset: boiling lysate, silica-column DNA kit, and/or plasmid preparation PCR reactions (25 μL) were performed using gene-specific primer pairs (Supplementary Table S6) under conventional cycling conditions (initial denaturation at ~95 °C, followed by 30–35 cycles of denaturation/annealing/extension, with a final extension at ~72 °C), along with appropriate positive and negative controls. The amplicons were resolved by agarose gel electrophoresis. An isolate was considered gene-positive if a band of the expected size was observed from any extraction method. Per-city PCR positivity counts for *blaOXA-23*, *blaNDM*, and *blaOXA-51-like* were also analyzed. To evaluate the diagnostic utility of rapid PCR against the reference phenotype, we computed the sensitivity of the composite marker (*blaOXA-23*, *blaNDM*) for predicting carbapenem resistance (reference: IMI BMD category) and reported Wilson 95% CIs and a between-city comparison (Fisher’s exact tests).

### Whole-genome sequencing (WGS), phylogenetic, and genomic typing

Genomic DNA was extracted from overnight cultures using a silica-column method (e.g., DNeasy Blood & Tissue Kit, Qiagen, Hilden, Germany) and quantified fluorometrically (Qubit dsDNA HS Assay, Thermo Fisher Scientific, Waltham, MA, United States). Libraries were prepared using Illumina DNA Prep (Illumina, San Diego, CA, United States) according to the manufacturer’s protocol and sequenced on an Illumina NovaSeq 6000 using an S2 flow cell, generating paired-end 2 × 150 bp reads. Raw reads were deposited to NCBI under BioProject PRJNA1402053.

### Genome assembly and genome annotation

Illumina paired-end reads were initially assessed for quality and trimmed using fastp (v1.0.1) with the parameters --cut_front, −-cut_tail, −-cut_mean_quality 30, −-length_required 50, and --detect_adapter_for_pe (Chen et al., 2018). Taxonomic composition was screened using Kraken2 (version 2.1.2) (Wood et al., 2019). Of the 71 phenotypically confirmed, non-duplicate *A. baumannii* isolates included in the BMD, VITEK II, and PCR analyses, 59 sequencing libraries passed WGS quality-control criteria and were retained for comparative genomic analysis. Libraries failing the <94% *A. baumannii* read-assignment threshold and/or downstream assembly/average nucleotide identity (ANI) quality filters were excluded from WGS analyses as a conservative measure to avoid contamination-driven or low-quality SNP calls. These exclusions do not reclassify the original clinical isolates as non-*A. baumannii*; rather, they indicate that the corresponding sequencing libraries were not suitable for high-resolution phylogenomic analysis. All 71 isolates remained included in the phenotypic and PCR analyses. Filtered reads were assembled *de novo* using SKESA (version 2.4.0) (Souvorov et al., 2018), and contigs <500 bp were removed using --min_contig 500. Species identity was confirmed by pairwise ANI comparison against *A. baumannii* AB5075 (GCF_000770605.1) using FastANI (version 1.33) (Jain et al., 2018), and assemblies with ANI < 97% were excluded from WGS-based downstream analyses. Assembly quality was assessed using QUAST (version 5.2.0) (Gurevich et al., 2013). All 71 isolates were retained for BMD, VITEK II, and PCR analyses. The retained genomes were annotated using Prokka (version 1.14.6) (Seemann, 2014), and gene-content/annotation completeness was assessed using BUSCO (version 6.0.0) (Simão et al., 2015).

### Variant calling and core-genome alignment

Illumina paired-end short-read WGS data were quality-trimmed to remove adapters and low-quality bases, then mapped to an *A. baumannii* GC2/sequence type 2 (ST2) reference genome (GenBank CP031380.1) using Snippy (version 4.6.0) with stringent thresholds (minimum depth ≥10×, base quality ≥30, and minimum allele frequency ≥0.9) to generate a core-genome SNP alignment across isolates. In parallel, assemblies were annotated with Prokka, and the resulting GFF3 files were analyzed using the Roary pan-genome (version 3.13.0) to define the pan-genome and construct a core-gene alignment comprising 2,539 genes present in ≥99% of the assembled isolates (Page et al., 2015). Maximum-likelihood phylogenies were inferred from the core-genome SNP alignment generated by reference-mapping (Snippy version 4.6.0) against an *A. baumannii* GC2/ST2 reference genome (GenBank CP031380.1). Putative recombinant regions were removed using Gubbins v3.3.0 (Croucher et al., 2015). SNP-only alignments (constant sites removed) were generated with snp-sites version 2.5.1 (Page et al., 2016). Trees were inferred in IQ-TREE2 (version 2.2.2) (Minh et al., 2019) using ModelFinder for model selection, ascertainment-bias correction (+ASC) where applicable, and 1,000 ultrafast bootstrap replicates for branch support. Trees were midpoint-rooted and annotated with metadata (city, Pasteur multilocus sequence typing [MLST], capsular locus [KL], and lipooligosaccharide outer-core locus [OCL] types).

### Genomic typing and locus annotation

Multilocus sequence typing (MLST) was assigned using the *A. baumannii* Pasteur scheme with the MLST tool (version 2.23.0) queried against the PubMLST database to define sequence types (STs) and major global clones (e.g., GC2/ST2 and GC1/ST1) in the PubMLST/BIGSdb database (Jolley et al., 2018). Capsular (KL) and lipooligosaccharide outer-core (OCL) loci were typed using Kaptive (version 3) with curated *A. baumannii* reference panels. Metadata tracks (city, ST, KL, and OCL) were displayed alongside the phylogeny to visualize population structure and geographic intermixing (Wyres et al., 2020; Stanton et al., 2025).

### Resistome profiling

AMR genes were called from assemblies using NCBI AMRFinderPlus (version 3.11.11; database version 2023-04-17.1). Detected antimicrobial resistance genes and point mutations were grouped by functional class, including carbapenemases and other beta-lactamases, aminoglycoside-modifying enzymes, fluoroquinolone targets, and efflux systems, to generate per-city heatmaps. Key markers included *blaOXA-23*, *blaNDM*, *armA*, aminoglycoside-modifying enzymes, *msrE*/*mphE*, *tet(B)*/*tetR*, *sul1*/*qacEΔ1*, and RND/MFS efflux components (Feldgarden et al., 2021).

### Pangenome analysis

Genomes were annotated with Prokka version 1.14.6, and the pangenome was inferred with Roary version 3.13.0 at 95% BLASTp identity and default MCL inflation = 1.5 (Multiple Alignment using Fast Fourier Transform [MAFFT] version 7 was used for core-gene alignment when applicable). Gene clusters were categorized as core (present in ≥58/59 genomes, ≥98%), soft-core (56–57/59, 95–97%), shell (8–55/59, 14–93%), and cloud (<8/59, ≤13%). Counts and category percentages are reported in Supplementary Figure S6.

### Method comparison and agreement

We used BMD as the reference. For each drug and city, we compared VITEK II categorical results with BMD and calculated overall agreement. We also reported standard error types when applicable: very major error (VME) = VITEK “Susceptible” vs. BMD “Resistant”; Major Error (ME) = VITEK “Resistant” vs. BMD “Susceptible”; Minor error = any discrepancy involving “Intermediate.”

For PCR, we assessed the ability of the composite marker (*blaOXA-23* or *blaNDM*) to identify carbapenem resistance using imipenem BMD as the reference. Given the high prevalence of resistance, we expect the PCR’s positive predictive value to be high in this setting. We conducted a sensitivity analysis to assess the impact of missing VITEK calls (no growth) on agreement metrics. For drug-city strata with unpaired isolates (where the VITEK result was missing while the BMD was available), we recalculated overall agreement, VME, ME, minor error, and *κ* after excluding these unpaired isolates. Main-text results report the paired analysis; sensitivity estimates are provided alongside.

### Statistical analysis

Analyses were performed in Python (pandas, NumPy, and SciPy). MICs were summarized by city as median (IQR), range, and proportions (e.g., % resistant) are presented with Wilson 95% confidence intervals (CIs). Between-city differences in resistance rates were tested with two-sided Fisher’s exact tests; *p*-values were not adjusted for multiple comparisons because the analysis was limited to a small, pre-specified set of drugs. Method agreement between VITEK II and BMD was summarized as percent agreement with Wilson 95% CIs; Cohen’s κ was estimated with bootstrap 95% CIs (2,000 resamples). CLSI error categories were applied: very major error (VME, BMD resistant, and VITEK susceptible), major error (ME, BMD susceptible, and VITEK resistant), and minor error (one intermediate and the other susceptible/resistant). Agreement analyses were restricted to paired VITEK–BMD results. PCR predictive performance (sensitivity, specificity, positive predictive value [PPV], and negative predictive value [NPV]) for the composite marker (*blaOXA-23* or *blaNDM*) vs. imipenem BMD is provided in Supplementary Table S5 with Wilson 95% confidence intervals.

## Results

### Study isolates and sources

We analyzed 71 multidrug-resistant (MDR) *A. baumannii* clinical isolates collected in Saudi Arabia from two sites (Riyadh, *n* = 36; Makkah, *n* = 35). Isolates were obtained from routine clinical specimens and represent a range of infection presentations. These two balanced city cohorts form the basis for all phenotype–genotype comparisons presented below.

#### Reference MICs by broth microdilution (CLSI)

**Figure 1:**
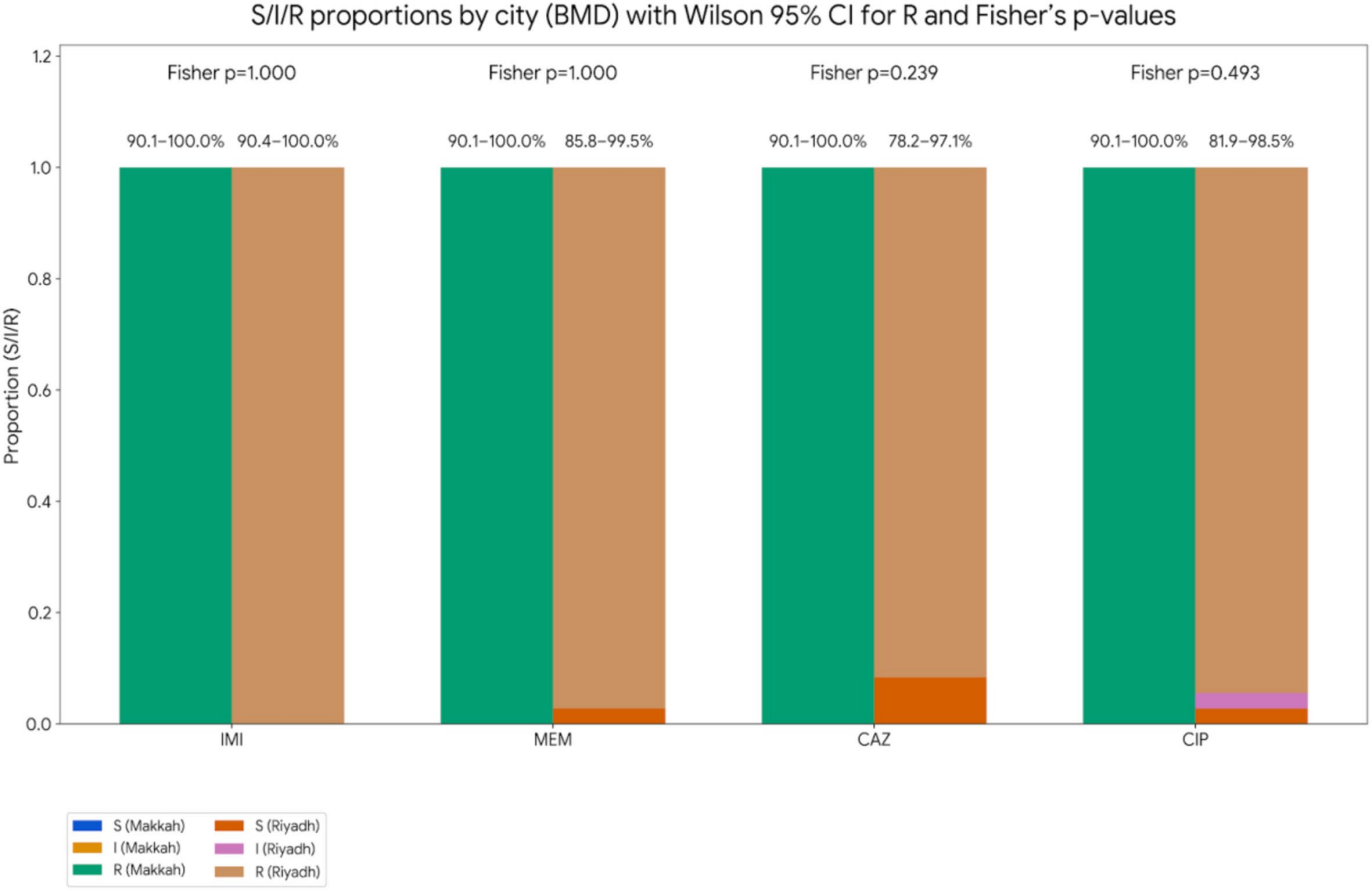
Stacked S/I/R proportions by city (reference BMD) with Wilson 95% CIs for the resistant proportion and Fisher’s exact tests’ *p*-values. For each drug (IMI, MEM, CAZ, CIP), stacked bars show the proportions of susceptible (S), intermediate (I), and resistant (R) isolates in Makkah (*n* = 35) and Riyadh (*n* = 36). Above each *R* bar is the Wilson 95% CI; above each drug pair is the two-sided Fisher’s exact test’s *p*-value (IMI 1.000; MEM 1.000; CAZ 0.239; CIP 0.493). Results confirm near-universal carbapenem resistance in both cities; slight differences in CAZ/CIP do not change interpretation. Counts and CIs are provided in Supplementary Table S1.

Carbapenem resistance by BMD (IMI/MEM) was near-universal (Makkah: 100%/100%; Riyadh: 100%/97.2%; Figure 1; Supplementary Table S1). In Makkah (*n* = 35), resistance was 100% to imipenem, meropenem, ceftazidime, and ciprofloxacin; median MICs (IQR) were IMI 1,024 (1,024–1,024) μg/mL, MEM 128 (128–192) μg/mL, CAZ 512 (256–512) μg/mL, and CIP 128 (128–128) μg/mL. In Riyadh (*n* = 36), resistance to imipenem remained 100%. Still, it was 97.2% for meropenem (1 susceptible at 0.5 μg/mL), 91.7% for ceftazidime (3 susceptible at ≤8 μg/mL), and 94.4% for ciprofloxacin (1 susceptible at 0.5 μg/mL; 1 intermediate at 2 μg/mL); median MICs were IMI 1024 (1,024–1,024) μg/mL, MEM 128 (64–256) μg/mL, CAZ 256 (128–320) μg/mL, and CIP 16 (16–64) μg/mL. Between-city differences in resistant proportions were not significant (Fisher’s exact test: IMI *p* = 1.000; MEM *p* = 1.000; CAZ *p* = 0.239; CIP *p* = 0.493), indicating a consistently high resistance burden across both sites (Supplementary Tables S3, S4).

### Automated phenotypes (VITEK II)

**Figure 2:**
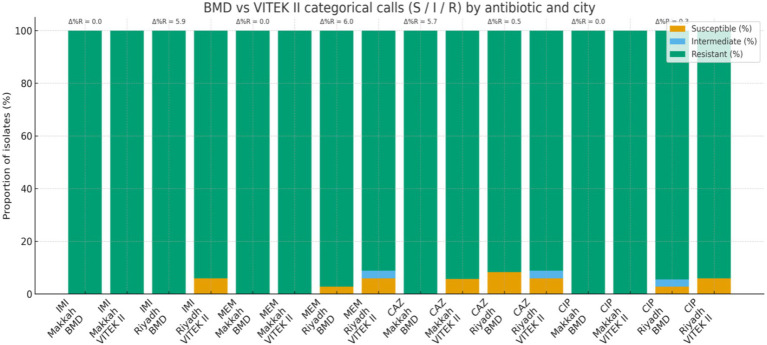
Agreement between reference BMD and VITEK II categorical calls (Susceptible/Intermediate/Resistant). For each antibiotic (IMI = imipenem, MEM = meropenem, CAZ = ceftazidime, CIP = ciprofloxacin) and each city (Makkah, Riyadh), stacked bars show the percentage of isolates classified as Susceptible (S), Intermediate (I), or Resistant (R) by each method (BMD or VITEK II). Each label on *x*-axis indicates Drug/City/Method, so each bar already indicates which method it represents. Above each BMD bar, we report *Δ*%R, defined as (BMD R% − VITEK II R%). A positive Δ%R indicates that BMD identified a higher proportion of isolates as Resistant than VITEK II for that antibiotic in that city. In Riyadh, minor differences partly reflect isolates that did not receive a valid VITEK II call (paired *n* = 34 vs. total *n* = 36). Comparison of BMD and VITEK II methods showing universal carbapenem resistance across isolates from Makkah and Riyadh. Minor discrepancies between the two methods do not alter the clinical conclusion of high drug resistance.

VITEK II categorical interpretations closely matched reference BMD, across all four antibiotics in both cities, with low very major error (VME) and major error (ME) rates and slight differences in the proportion of resistant isolates (*Δ*%R). In Makkah, imipenem, meropenem, ciprofloxacin, cefepime, and trimethoprim/sulfamethoxazole were almost uniformly resistant (≥94%), with small susceptible pockets to ceftazidime (2/35) and gentamicin (2/35); tigecycline split across susceptible/intermediate categories (13 S, 20 I, and R). In Riyadh, carbapenem resistance remained very high (imipenem 32/34 R with 2 S; meropenem 31 R, 1 I, and 2 S), while non-carbapenem classes showed greater susceptible fractions, notably gentamicin (11 S) and tigecycline (30 S, 1 I, and 3 R) (Figure 2). These categorical trends align with the BMD medians and underscore the limited therapeutic window against these isolates (Figure 2). Detailed VITEK II categorical results are provided in Supplementary Table S2.

### Method comparison and agreement

We compared VITEK II against BMD per drug and city (Table 1). Where BMD categories were homogeneous (e.g., 100% R), exact VME/ME were computed; we report categorical agreement, VME/ME/minor error rates, and Cohen’s *κ* (Table 1). In strata with missing VITEK calls due to no growth (e.g., Riyadh IMI, n paired = 35 of 36), agreement metrics were unchanged within rounding when excluding unpaired isolates: VME 5.7% (2/35); ME 0%; minor error 0%; κ not estimable where marginals collapsed.

**Table 1:** Agreement of VITEK II categorical interpretations vs. BMD (reference) by city and drug.

City	Drug	Paired *n*	Agreement % (95% CI)	VME *n*/*N* (%)	ME *n*/*N* (%)	Minor *n*/*N* (%)	Cohen’s *κ* (95% CI)
Makkah	IMI	35	100.0 (90–100)	0/35 (0.0)	NA	0/35 (0.0)	NA
Makkah	MEM	35	100.0 (90–100)	0/35 (0.0)	NA	0/35 (0.0)	NA
Makkah	CAZ	35	94.3 (81–98)	2/35 (5.7)	NA	0/35 (0.0)	0.00 (0.00–0.00)
Makkah	CIP	35	100.0 (90–100)	0/35 (0.0)	NA	0/35 (0.0)	NA
Riyadh	IMI	34	94.1 (81–98)	2/34 (5.9)	NA	0/34 (0.0)	0.00 (0.00–0.00)
Riyadh	MEM	34	94.1 (81–98)	1/33 (3.0)	0/1 (0.0)	1/34 (2.9)	0.48 (0.00–1.00)
Riyadh	CAZ	34	94.1 (81–98)	1/32 (3.1)	0/2 (0.0)	1/34 (2.9)	0.57 (0.00–1.00)
Riyadh	CIP	34	94.1 (81–98)	1/32 (3.1)	0/1 (0.0)	1/34 (2.9)	0.48 (−0.03–1.00)

BMD, broth microdilution (reference); VME, very major error (BMD R; VITEK S); ME, major error (BMD S; VITEK R); Minor error, either method I while the other is S or R. 95% CI for agreement from Wilson; κ 95% CI by bootstrap (2,000 resamples). Breakpoints: CIP ≤1/2/≥4; CAZ ≤8/16/≥32; MEM ≤2/4/≥8; IMI ≤2/4/≥8 (S/I/R, μg/mL).

### Targeted PCR genotyping (blaOXA-23, blaNDM, and blaOXA-51-like)

**Figure 3:**
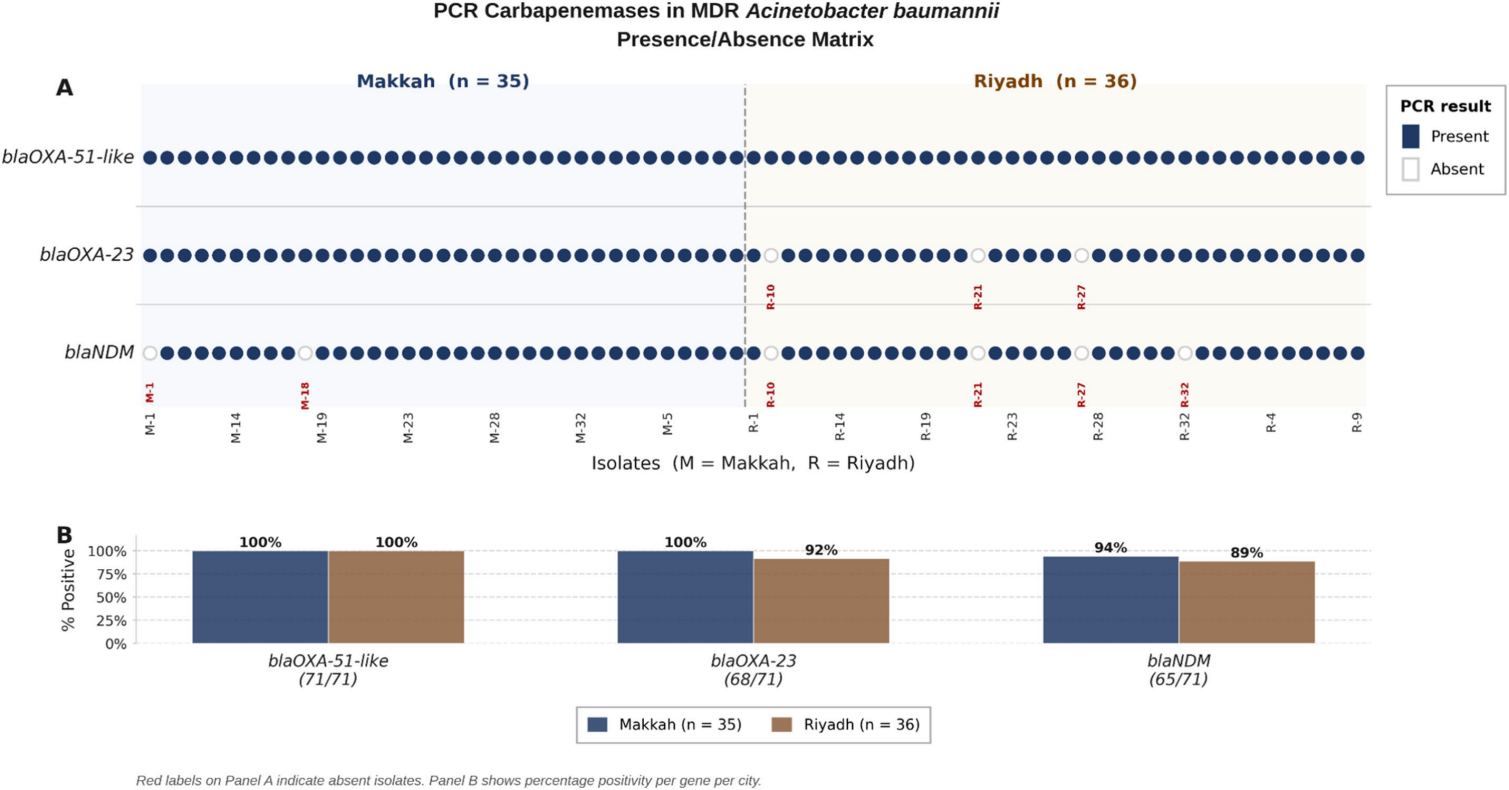
PCR presence/absence of carbapenemase genes in multidrug-resistant *Acinetobacter baumannii* (*n* = 71; Makkah *n* = 35, Riyadh *n* = 36). **(A)** Each dot represents one clinical isolate; filled (navy) = gene detected; open (grey) = gene absent. Absent isolates are labeled in red. Each row represents one PCR target: *bla*OXA-*51-like* (intrinsic marker, 71/71), *bla*OXA-*23* (acquired carbapenemase, 68/71), and *bla*NDM (metallo-*β*-lactamase, 65/71). **(B)** Per-city percentage positivity for each gene (*bla*OXA-*23*: 100% Makkah, 92% Riyadh; *bla*NDM: 94% Makkah, 89% Riyadh). The near-universal detection of *bla*OXA-*23*, often with co-detection of *bla*NDM, is consistent with the high carbapenem-resistance burden observed in the reference BMD and supports the use of focused PCR as a same-day rule-in tool in this setting.

Targeted PCR demonstrated universal *blaOXA-51-like* detection and a carbapenemase backbone dominated by *blaOXA-23* with substantial *blaNDM* co-circulation (Figure 3). *blaOXA-23* was detected in 95.8% of isolates overall (Makkah 35/35; Riyadh 33/36), and *blaNDM* in 91.5% (Makkah 33/35; Riyadh 32/36). Using the combined marker “*blaOXA-23* or *blaNDM* positive” to predict carbapenem resistance (reference: imipenem BMD), sensitivity was 95.8% overall, 100% in Makkah (35/35; Wilson 95% CI ≈ 90–100%) and 91.7% in Riyadh (33/36; Wilson 95% CI ≈ 79–98%) with no significant between-city difference by Fisher’s exact test (*p* = 0.239, data not shown) (Table 2). Diagnostic performance of the composite carbapenemase PCR (*blaOXA-23* or *blaNDM*) against imipenem BMD is summarized by city in Supplementary Table S5 (sensitivity with Wilson 95% CIs; specificity/NPV not estimable due to no imipenem-susceptible isolates).

**Table 2:** PCR positivity by city.

City	*N*	*blaOXA-51-like* (+)	*blaOXA-23* (+)	*blaNDM* (+)
Makkah	35	35	35	33
Riyadh	36	36	33	32

A resistome heatmap (Supplementary Figures S4, S5) revealed broadly similar AMR gene-family profiles in Makkah and Riyadh, characterized by OXA-type *β*-lactamases, quinolone target determinants (*gyrA*/*parC*), aminoglycoside-modifying enzymes, and abundant RND/MFS efflux systems, consistent with the observed MDR phenotype. These data support the use of same-day PCR as a high-positive-predictive-value (PPV) rule-in tool in this *blaOXA–23*–rich setting (Supplementary Table S5), while Table 3 summarizes the per-city sensitivity of this combined PCR marker (*blaOXA-23 or blaNDM*) for carbapenem resistance. Phenotype–genotype concordance was strongest for carbapenem resistance: the near-universal imipenem/meropenem resistance observed by BMD and VITEK II corresponded to the high prevalence of *blaOXA-23*, frequently accompanied by *blaNDM*, and to WGS-based detection of additional β-lactamase and efflux-associated determinants. Importantly, three Riyadh isolates were imipenem-resistant by BMD but negative for the composite *blaOXA-23*/*blaNDM* PCR marker, indicating that focused PCR is best interpreted as a rapid rule-in tool rather than a rule-out assay. For non-carbapenem agents, fluoroquinolone and aminoglycoside resistance patterns were broadly consistent with detection of quinolone target determinants, aminoglycoside-modifying enzymes/*armA*, and multidrug efflux systems; however, genotype-to-phenotype prediction was not assumed where expression, regulatory mutations, or breakpoint limitations may influence categorical AST interpretation.

**Table 3:** PCR sensitivity of *blaOXA-23* or *blaNDM* for carbapenem resistance (reference: imipenem BMD).

City	*n*	PCR positive (*blaOXA-23* or *blaNDM*)	Sensitivity (95% CI)
Makkah	35	35	100.0% (90.1–100.0)
Riyadh	36	33	91.7% (78.2–97.1)

PCR, polymerase chain reaction; BMD, broth microdilution; CI, confidence interval.

**Table 4:** Core-genome SNP micro-clusters (≤10 SNPs; cluster size ≥2) and city composition among WGS genomes (*n* = 59).

Cluster ID	Size	City	Maximum SNP	IPC interpretation
CL_1	22	Makkah = 22	≤10	Putative local/shared-source micro-cluster; trigger outbreak investigation and enhanced IPC bundle.
CL_2	5	Riyadh = 5	≤10	Putative local/shared-source signal; review temporal–spatial links and reinforce IPC.
CL_3	3	Riyadh = 3	≤10	Putative local/shared-source signal; review temporal–spatial links and reinforce IPC.
CL_4	3	Riyadh = 3	≤10	Putative local/shared-source signal; review temporal–spatial links and reinforce IPC.
CL_5	2	Makkah = 2	≤10	Putative local/shared-source signal; review temporal–spatial links and reinforce IPC.
CL_6	2	Riyadh = 2	≤10	Putative local/shared-source signal; review temporal–spatial links and reinforce IPC.
CL_7	2	Riyadh = 2	≤10	Putative local/shared-source signal; review temporal–spatial links and reinforce IPC.
CL_8	2	Riyadh = 2	≤10	Putative local/shared-source signal; review temporal–spatial links and reinforce IPC.

Micro-clusters were defined using pairwise distances from the recombination-filtered core-genome SNP alignment. Only multi-isolate micro-clusters (size ≥2) are shown; the remaining 18/59 genomes were singletons at the ≤10-SNP threshold. All multi-isolate micro-clusters were single cities (Mixed = No). The largest Makkah micro-cluster contained 22 genomes; the largest Riyadh micro-cluster contained five genomes.

### WGS phylogenetic, MLST, KL/OCL, and resistome

**Figure 4:**
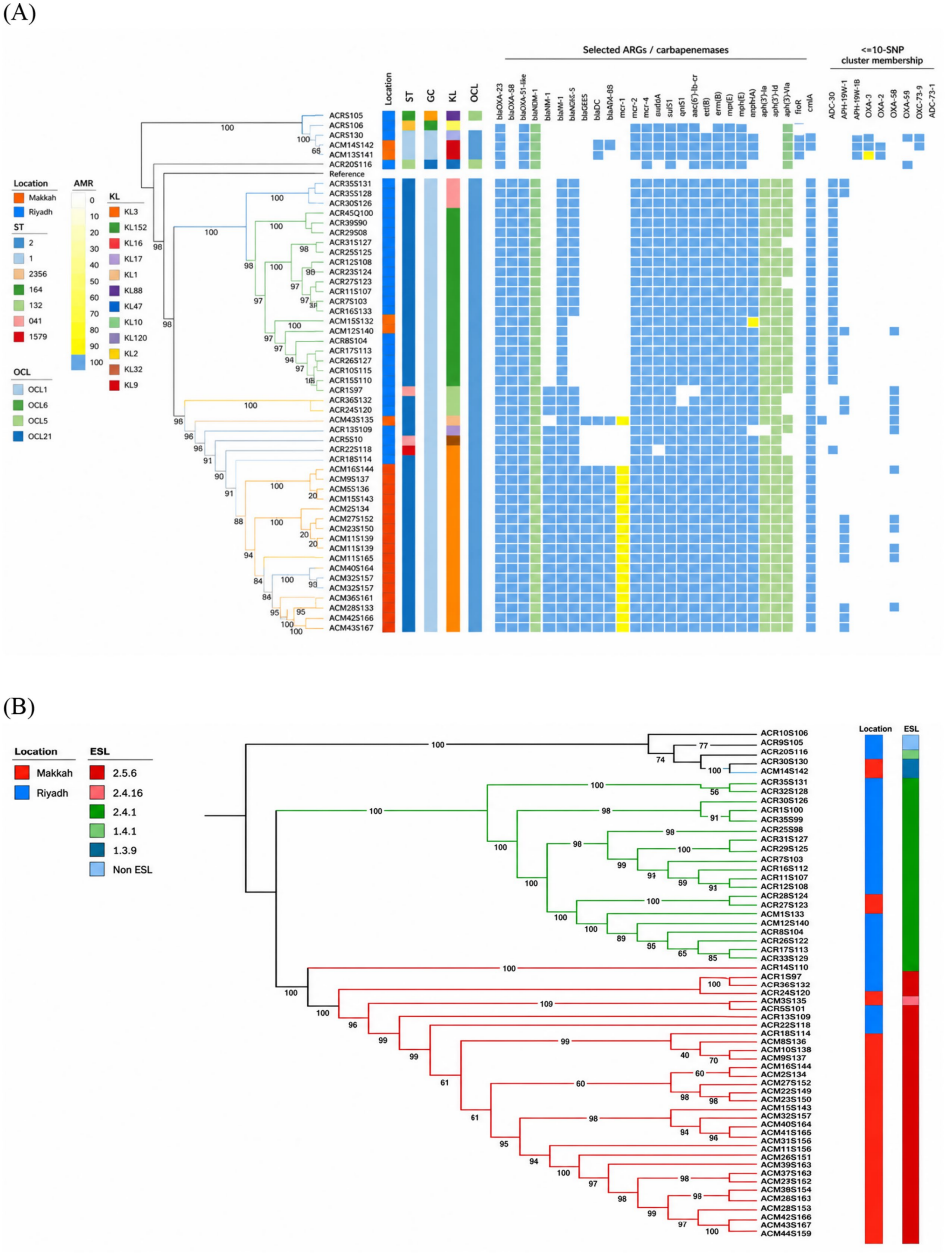
Recombination-filtered core-genome phylogeny and whole-genome alignment (WGA) comparison. **(A)** Maximum-likelihood phylogeny inferred from recombination-filtered core-genome SNP alignment (Snippy version 4.6.0 for variant calling; Gubbins version 3.3.0 for recombination filtering; IQ-TREE2 for maximum-likelihood inference). Tip metadata tracks indicate city/location (Makkah = red; Riyadh = blue), Pasteur ST/global clone assignment, KL (capsular locus) type, OCL (lipooligosaccharide outer-core locus) type, selected ARG/carbapenemase markers including *blaOXA-23* and *blaNDM*, and ≤10-SNP micro-cluster membership (cluster labels shown). Branch colors reflect ultrafast bootstrap support (0–100%). The dominant lineage is GC2/ST2 (international clone 2 [IC2]), with a minor non-GC2 branch (including GC1/ST1 and related STs). Makkah and Riyadh isolates are interleaved across the major clades, indicating cross-city regional circulation. Cluster labels identify putative recent local transmission or shared-source groups that are single-city in composition, supporting local Infection Prevention and Control (IPC) investigation triggers rather than confirmed direct transmission. Sensitivity analyses at ≤5 and ≤25 SNPs gave the same qualitative interpretation; cluster membership is summarized in Table 4. **(B)** WGA tree without recombination filtering, reproducing the same major splits and city-interleaved topology, confirming that the observed clusters are not artifacts.

The recombination-filtered core-genome ML tree shows a polyclonal, yet structured population dominated by GC2/ST2, with a minor non-GC2 branch (including GC1/ST1 and related STs) (Figure 4A). Makkah and Riyadh isolates are interleaved across the main clades, indicating cross-city circulation rather than city-specific lineages. This pattern is not attributable to tree reconstruction artifacts: a reference-mapped whole-genome alignment (WGA) tree reproduced the same major splits and city interleaving (Figure 4B), and recombination filtering shortened branches without altering the topology or cluster membership. Within clades, we observe small city-weighted micro-clusters (short internal branches), consistent with local amplification; however, each city contributes isolates to multiple branches, underscoring epidemiologic connectivity. MLST agrees with the topology (ST2 predominant; ST1/mixed in the secondary branch). KL and OCL types called by Kaptive (confidence ≥ good) broadly co-segregate with clades and are shared between cities (e.g., KL3/KL152/KL120/KL17 and OCL1/OCL5/OCL15/OCL21/OCL6; detailed counts are shown in Supplementary Figure S3), consistent with a GC2/ST2-dominant background rather than city-specific capsular lineages. The resistome is likewise shared across cities—widespread *blaOXA-23* with *armA*, aminoglycoside-modifying enzymes, *msrE/mphE*, *tet(B)/tetR*, class-1 integron markers (*sul1* and *qacEΔ1*), and RND/MFS efflux systems (e.g., *adeABC*/*adeIJK*/*adeFGH*); *mcr* was not detected. Per-isolate profiles in the resistome heatmap mirror this pattern and show no geography-restricted resistance family (Supplementary Figures S4, S5), aligning with the MDR phenotype and the high PPV of our focused carbapenemase PCR rule-in (Supplementary Table S5).

## Pangenome structure

Across the 59 genomes, the pangenome comprised 6,869 gene clusters: core 2,539 (37%), soft-core 263 (3.8%), shell 1,126 (16%), and cloud 2,941 (43%), indicating a substantial accessory genome consistent with *A. baumannii* gene flux and the GC2-dominant population structure (Supplementary Figure S6).

## Discussion

This dual-city analysis of 71 MDR *A. baumannii* isolates generated three operational findings: (i) Reference BMD confirmed an extreme resistance burden; carbapenem resistance was essentially universal in both cities, supporting immediate IPC action when CRAB is suspected. (ii) Compared with BMD, VITEK II showed high categorical agreement (94–100% across drug–city strata) but non-zero very major error rates for carbapenems (up to 5.9%), meaning automated “susceptible” calls can occur in high-prevalence CRAB settings and should be reflex-confirmed when they would change therapy or cohorting decisions. (iii) A focused same-day PCR rule-in (composite *blaOXA-23* or *blaNDM*) demonstrated high sensitivity for BMD-defined carbapenem resistance (100% in Makkah; 91.7% in Riyadh), while WGS showed GC2/ST2 predominance with interleaved clades across cities (regional circulation) but predominantly single-city ≤10-SNP micro-clusters (0 mixed-city clusters), indicating putative recent local transmission or shared-source signals.

Rapid PCR detected *blaOXA-23* and/or *blaNDM* in most isolates, with high sensitivity compared with BMD for predicting carbapenem resistance, yielding a PPV of 100% at the observed high prevalence (Supplementary Table S5). WGS resolved a polyclonal but structured population dominated by GC2/ST2, with city-interleaved tips and KL/OCL types co-segregating with clades; no *mcr* genes were observed. Together, these data indicate cross-city circulation with city-weighted micro-clusters, supporting integrated PCR–phenotype–WGS surveillance. This enables same-day PCR “rule-in” isolation and routine (e.g., weekly) WGS cluster scans to prioritize unit-level IPC interventions. Together, Supplementary Figures S1–S3 reinforce our operational ≤10-SNP micro-cluster rule, support putative recent local transmission or shared-source amplification signals that are primarily single-city, with minimal cross-city mixing, and show KL/OCL diversity without geography-restricted types, consistent with a GC2/ST2-dominant population structure. The agreement between the WGA tree and the recombination-filtered core-genome SNP tree increases confidence that the observed clusters are phylogenetically robust and not artifacts. In contrast, core-genome distances provide an operational definition of ≤10 SNPs to prioritize IPC actions (with sensitivities of ≤5 and ≤25 SNPs).

Our findings align with the global status of CRAB as a WHO critical-priority pathogen, reflecting sustained high resistance, limited therapeutic options, and excess mortality in severe infections (Castanheira et al., 2023; Sorbello et al., 2023; World Health Organization, 2024). The near-universal carbapenem resistance observed is consistent with international syntheses in which OXA-type carbapenemases predominantly *blaOXA-23* anchor resistance in high-risk GC2/ST2 (international clone 2 [IC2]) lineages, with regional contributions from *blaNDM* in some networks (Zowawi et al., 2015; Castanheira et al., 2023). Within Saudi Arabia, national reviews and multicenter observations consistently document endemic CRAB with ICU concentrations and adverse outcomes; our Riyadh/Makkah phenotype distribution therefore reflects the national signal rather than an anomaly (Ibrahim, 2019; Yasir et al., 2022; Thabit et al., 2025).

Using BMD as the reference, we compared VITEK II categorical calls. We found strong agreement for carbapenems but greater variability for non-carbapenem agents, mirroring recent evaluations that show drug-specific performance limits for automated systems and emphasize the value of BMD confirmation in high-resistance settings (Ananda et al., 2024; Forstner et al., 2024; Kim et al., 2025). Our *Δ*%R annotations (BMD vs. VITEK II) make these differences explicit for stewardship and can be operationalized (e.g., reflex BMD for edge-case categories).

The composite “*blaOXA-23* or *blaNDM*” PCR marker displayed high sensitivity for carbapenem resistance in our set, consistent with the global predominance of *blaOXA-23* and real-world performance of rapid assays for *A. baumannii* carbapenemases (Castanheira et al., 2023; World Health Organization, 2024; Sati and Laxminarayan, 2025). In a high-prevalence context, PPV is strong; the few PCR-negative/phenotype-positive cases reinforce the need to maintain reference MICs and to consider non-enzymatic mechanisms (e.g., efflux and permeability changes).

Recombination-filtered core-SNP phylogenetics resolved a GC2/ST2-dominated population with a minor non-GC2 branch (including GC1/ST1 and related STs) and interleaved tips from both cities, indicating regional movement rather than strict geographic partitioning. This mirrors multi-site genomic studies in which GC2 disseminates across hospitals and borders, producing clonal “waves” that persist or re-seed units over time (Castanheira et al., 2023). KL/OCL overlays, assigned using curated Kaptive databases, co-segregated with clades, consistent with contemporary catalogs that capture prevalent KL repertoires and show OCL1 dominance across public genomes (Wyres et al., 2020; Sorbello et al., 2023). Environmental reservoirs and micro-clusters. Evidence from an ICU in Saudi Arabia shows that environmental CRAB and contemporary patient isolates share the same sequence types and minimal core-genome SNP distances, demonstrating direct genetic relatedness and environmental persistence within the built environment (e.g., high-touch surfaces and sinks) (Yasir et al., 2022). These data argue that the ICU environment can act as a sustained reservoir, with recurrent seeding into patients and equipment. Our phylogeny shows small, city-weighted micro-clusters superimposed on an interleaved backbone; taken together, this pattern is most consistent with putative local transmission or shared-source amplification events rather than distinct city-specific lineages. Confirmation of direct transmission chains will require linked temporal and ward-level epidemiological data. Consequently, reservoir-focused interventions (sink/pipe hygiene, device re-processing, enhanced terminal cleaning, and cohorting) are likely to have an outsized impact.

Taken together, the WGS data explain why carbapenem resistance is nearly universal phenotypically and why the same *blaOXA-23* ± *blaNDM* backbone is detectable by rapid PCR in both cities. Both hospitals are drawing from the same high-risk GC2/ST2 reservoir rather than from unrelated local clones.

The resistome we overlaid--*blaOXA-23* (with or without *blaNDM*), frequent *armA*/aminoglycoside-modifying enzymes, *msrE/mphE, tet(B)/tetR, sul1/qacEΔ1*, and ade-family RND efflux systems is prototypical for CRAB and mechanistically explains broad MDR beyond carbapenems (Castanheira et al., 2023; Shi, 2024; Zack et al., 2024; Xie et al., 2025). Experimental and clinical studies continue to implicate *AdeABC/AdeIJK/AdeFGH* overexpression in diminished susceptibility (e.g., tigecycline) and broader MDR phenotypes (Zack et al., 2024; Xie et al., 2025). The absence of *mcr* (mobilized colistin resistance gene) in our isolates is reassuring; nonetheless, accurate colistin testing remains crucial, and BMD remains the reference for polymyxins in guidelines and method comparisons (Ananda et al., 2024; Forstner et al., 2024; Kim et al., 2025).

Positioning within Saudi Arabian and regional literature, our dual-city collection sits on a continuum defined by the GCC molecular survey (*blaOXA-23* dominance), national reviews from Saudi Arabia (high CRAB prevalence and ICU burden), and environmental WGS studies (clinical–environment relatedness) (Zowawi et al., 2015; Ibrahim, 2019; Yasir et al., 2022). A previous study from Saudi Arabia by Alyamani on ESBLs in *A. baumannii* documented *β*-lactamase diversity among *A. baumannii.* It highlighted the genetic heterogeneity that plausibly coexists with the *blaOXA-23-centric* carbapenem-resistance backbone now observed in our cohort (Alyamani et al., 2015). Across regions, some centers report *blaNDM-dominant* outbreaks, whereas others remain *blaOXA-23-dominant*; our high *co-occurrence of blaOXA-23* and *blaNDM* resembles settings in which co-producers are increasingly encountered, although proportions vary over time and by location (Castanheira et al., 2023; Ababneh and Al-Sweedan, 2025).

Similar to multi-country reports, we observed GC2 predominance, *blaOXA-23* carriage, and inter-facility circulation (Zowawi et al., 2015; Castanheira et al., 2023; Ababneh and Al-Sweedan, 2025). Differences include an interleaved cross-city phylogeny, contrasting with studies of facility-constrained clades during contained outbreaks; this suggests active regional flow (referrals, shared care pathways, mass gatherings) and argues for coordinated, region-level IPC rather than hospital-only approaches. Regarding methods, our VITEK II–BMD carbapenem agreement was high. Still, we echo recent studies showing that agent-dependent agreement declines for other classes, supporting local verification and reflex testing algorithms (Ananda et al., 2024; Forstner et al., 2024; Kim et al., 2025).

For a critical-priority pathogen with near-universal carbapenem resistance, same-day carbapenemase PCR accelerates isolation, cohorting, and early therapy decisions while confirmatory BMD proceeds. WGS adds transmission resolution and contextualizes KL/OCL diversity relevant to immunotherapies and phage-based strategies. Given the city-interleaved pattern we observed, shared alerts, joint investigations, and environmental decontamination in hot-spot units across the region are advisable.

### Implications for infection prevention and control (IPC): outbreak response and triage

(1) Same-day triage (0–8 h): PCR rule-in triggers immediate containment. Perform targeted PCR for *blaOXA-23* and *blaNDM* as soon as *A. baumannii* is isolated from any high-risk unit (e.g., ICU, burns, and transplant) or any patient meeting MDR Gram-negative isolation criteria. A positive result (*blaOXA-23* and/or *blaNDM*) should be treated as presumptive CRAB. It should trigger immediate contact precautions, cohorting (or single-room placement when feasible), dedicated equipment, and rapid notification of IPC and antimicrobial stewardship to support timely patient placement and empiric therapy decisions.

(2) Confirmatory phenotype (18–24 h): reflex reference MICs for decision-changing AST calls. Use BMD as the reference method for carbapenems when therapy, de-escalation, or discontinuation of precautions depends on categorical results. Any automated carbapenem “susceptible” call in a high-prevalence CRAB setting should be reflexively confirmed by BMD before changing isolation/cohorting decisions, thereby mitigating the clinical impact of rare but high-consequence very major errors.

(3) Genomic escalation (triggered/rolling): WGS micro-cluster surveillance as the outbreak alarm. Sequence CRAB isolates on a rolling schedule and immediately for suspected temporal–spatial clusters. Operationalize the ≤10 core-SNP threshold as an outbreak trigger: when a new isolate falls within an existing micro-cluster, initiate an outbreak investigation (case finding, unit mapping, and exposure review), reinforce cohorting and environmental cleaning, and consider targeted point-prevalence screening and environmental sampling (especially sinks/drains and shared devices) based on local IPC policy (Table 4).

(4) Operational shift: from retrospective description to real-time containment. Together, same-day PCR provides rapid rule-in triage, BMD safeguards against decision-changing automated AST errors, and WGS supplies an objective outbreak trigger and monitoring metric. Implementing this integrated characterization enables faster containment of CRAB transmission in high-burden hospitals and provides auditable criteria for escalation and de-escalation of IPC measures.

### Study limitations

This study included 71 MDR *A. baumannii* isolates from two tertiary hospitals (Makkah and Riyadh) over a limited timeframe; therefore, it may not capture the full diversity of CRAB circulating in other regions or settings. Isolates were obtained through routine clinical workflows (treatment-seeking patients), and we did not systematically sample colonization or the environment; thus, silent reservoirs and transmission pathways may be underestimated. Our rapid PCR targeted *bla*OXA-23 and *blaNDM*, which explained nearly all carbapenem resistance here. Still, rare/emerging carbapenemases (e.g., *blaOXA-58* or non-NDM MBLs) could be missed without broader assays or WGS-based screening. We did not perform phenotypic colistin testing nor long-read/plasmid-resolved assemblies, so while no *mcr* was detected, other colistin resistance mechanisms or plasmid contexts cannot be excluded. Isolate de-identification was required by the institutional ethics framework governing this retrospective study, and ward-level patient movement records and high-resolution collection dates were not available for all isolates in the analyzable dataset. Consequently, the ≤10-SNP groups reported here should be interpreted as putative recent-transmission or shared-source signals suitable for IPC investigation, rather than as definitive proof of patient-to-patient transmission chains. Finally, detailed clinical outcomes and infection-control data were not collected, further limiting direct linkage of genomic clusters to specific transmission events. Future prospective surveillance should link WGS to collection date, ward, admission/discharge data, and environmental sampling to confirm transmission routes. Despite these constraints, the concordant BMD, PCR, and WGS findings, and their agreement with national/international reports, support the robustness and generalizability of our main conclusions.

## Conclusion

Carbapenem-resistant *A. baumannii* remains a critical threat to patient safety and hospital infection prevention, and understanding both diagnostic performance and transmission structure is essential for timely control. In this dual-city investigation of MDR *A. baumannii* from Makkah and Riyadh, we combined reference broth microdilution (BMD), routine VITEK II susceptibility testing, targeted same-day carbapenemase PCR (*blaOXA-23* and *blaNDM*), and whole-genome sequencing (WGS)-based phylogenetics to link clinical decision-making with real-time surveillance. BMD confirmed an extreme carbapenem resistance burden, while VITEK II showed high agreement but non-zero very major errors, supporting reflex MIC confirmation for decision-changing susceptible calls. Targeted PCR provided a practical rule-in triage signal, and WGS demonstrated GC2/ST2 predominance with predominantly local≤10-SNP micro-clusters, consistent with ongoing local transmission and supporting an IPC model in which rapid PCR containment is paired with genomic outbreak triggering and continuous micro-cluster surveillance. Future studies should expand sampling density and duration, integrate epidemiologic metadata, and evaluate prospective workflows (including costs and turnaround times) to optimize thresholds and implementation for routine hospital IPC.

## Data Availability

Raw Illumina paired-end sequencing reads for all 59 whole-genome-sequenced isolates have been deposited at NCBI under BioProject accession PRJNA1402053. Assembled genome sequences and associated metadata are available through the same BioProject. Minimum inhibitory concentration data and VITEK II categorical outputs are available from the corresponding author upon reasonable request.
