# Supplementary material for: Phenotypic, PCR, and whole-genome sequencing characterization of carbapenem-resistant Acinetobacter baumannii in Makkah and Riyadh, Saudi Arabia: AST discordance and local genomic micro-clusters
Source: Front Microbiol. 2026 Jun 22;17:1845444. doi: 10.3389/fmicb.2026.1845444 (PMC13333605; doi:10.3389/fmicb.2026.1845444)
Supplement: Supplementary file 1 [file Table_1.DOCX]

**Supplementary Materials**

**Table S1-1.** Minimum Inhibitory Concentration (MIC) Determination in *A. baumannii* clinical isolates from the Makkah Region.

| *A. baumannii* | Ciprofloxacin  R ≥ 4 µg/ml | Ceftazidime  R ≥ 32µg/ml | Meropenem  R≥ 8 µg/ml | Imipenem  R ≥ 8 µg/ml |
| --- | --- | --- | --- | --- |
| 1 | 8 | ≥1024 | 128 | ≥1024 |
| 2 | 128 | 1024 | 256 | ≥1024 |
| 3 | 256 | 128 | 128 | ≥1024 |
| 8 | 128 | 256 | 128 | ≥1024 |
| 9 | 128 | 256 | 256 | ≥1024 |
| 10 | 128 | 512 | 64 | ≥1024 |
| 11 | 128 | 512 | 128 | ≥1024 |
| 12 | 64 | 128 | 256 | ≥1024 |
| 13 | 8 | 256 | 32 | ≥1024 |
| 14 | 8 | 128 | 128 | ≥1024 |
| 15 | 128 | 512 | 128 | ≥1024 |
| 16 | 128 | 1204 | 64 | ≥1024 |
| 17 | 128 | 512 | 128 | ≥1024 |
| 18 | 128 | 512 | 128 | ≥1024 |
| 19 | 128 | 512 | 128 | ≥1024 |
| 21 | 128 | 1204 | 128 | ≥1024 |
| 22 | 128 | 256 | 256 | ≥1024 |
| 23 | 128 | 512 | 128 | ≥1024 |
| 26 | 128 | 512 | 256 | ≥1024 |
| 27 | 128 | 256 | 128 | ≥1024 |
| 28 | 128 | 256 | 128 | ≥1024 |
| 29 | ≥1024 | 256 | 128 | ≥1024 |
| 30 | 128 | 256 | 128 | ≥1024 |
| 31 | 128 | 256 | 256 | ≥1024 |
| 32 | 64 | ≥1024 | 128 | ≥1024 |
| 33 | 128 | 512 | 128 | ≥1024 |
| 34 | 128 | 512 | 128 | ≥1024 |
| 35 | 128 | 512 | 64 | ≥1024 |
| 36 | 128 | 512 | 64 | ≥1024 |
| 37 | 128 | 512 | 128 | ≥1024 |
| 39 | 128 | 32 | ≥1024 | ≥1024 |
| 40 | 64 | 512 | ≥1024 | ≥1024 |
| 41 | 64 | 512 | ≥1024 | ≥1024 |
| 42 | 128 | 512 | 32 | ≥1024 |
| 43 | 64 | 512 | 128 | ≥1024 |
| Resistance ratio | 100% R | 100% R | 100% R | 100% R |

**Table S1-2.** Minimum inhibitory concentration (MIC) determination in *A. baumannii* clinical isolates from Riyadh region.

| *A. baumannii* | Ciprofloxacin  R ≥ 4 µg/ml | Ceftazidime  R ≥ 32µg/ml | Meropenem  R ≥ 8 µg/ml | Imipenem  R ≥ 8 µg/ml |
| --- | --- | --- | --- | --- |
| 1 | 128 | 512 | 64 | ≥1024 |
| 2 | 8 | 256 | 64 | ≥1024 |
| 3 | 8 | 128 | 64 | ≥1024 |
| 4 | 8 | 128 | 32 | ≥1024 |
| 5 | 128 | 1024 | 32 | ≥1024 |
| 6 | 64 | 32 | 64 | ≥1024 |
| 7 | 16 | 256 | 64 | ≥1024 |
| 8 | 16 | 64 | 16 | ≥1024 |
| 9 | 64 | **8** | 64 | ≥1024 |
| 10 | 8 | 64 | 8 | ≥1024 |
| 11 | 16 | 128 | 256 | ≥1024 |
| 12 | 32 | 128 | 256 | ≥1024 |
| 13 | 128 | 512 | 256 | ≥1024 |
| 14 | 16 | 128 | 128 | ≥1024 |
| 15 | 256 | 128 | 256 | ≥1024 |
| 16 | 16 | 256 | 256 | ≥1024 |
| 17 | 16 | 128 | 128 | ≥1024 |
| 18 | 64 | 512 | 256 | ≥1024 |
| 19 | 16 | 128 | 128 | ≥1024 |
| 20 | 32 | 512 | 32 | 8 |
| 21 | 0.5 | 8 | 0.5 | 128 |
| 22 | 128 | 512 | 256 | ≥1024 |
| 23 | 16 | 256 | 256 | ≥1024 |
| 24 | 64 | 512 | 128 | ≥1024 |
| 25 | 32 | 512 | 256 | ≥1024 |
| 26 | 1024 | 128 | 64 | ≥1024 |
| 27 | 32 | 128 | 128 | ≥1024 |
| 28 | 32 | 128 | 128 | ≥1024 |
| 29 | 8 | 256 | 128 | ≥1024 |
| 30 | 16 | 256 | 128 | ≥1024 |
| 31 | 32 | 256 | 512 | ≥1024 |
| 32 | 16 | 256 | 128 | ≥1024 |
| 33 | 16 | 256 | 128 | ≥1024 |
| 34 | **2** | **8** | 64 | ≥1024 |
| 35 | 16 | 256 | 256 | ≥1024 |
| 36 | 64 | 1024 | 256 | ≥1024 |
| Resistance ratio | R 94% (34/36)  S 3% (1/36)  I 3% (1/36) | R 91.50% (33/36)  S 8.5% (3/36) | R 97% (35/36)  S 3% (1/36) | R 100%  (36/36) |

**Table S2**. Diagnostic performance of composite carbapenemase PCR (*blaOXA*‑23 OR *blaNDM*) versus imipenem BMD (city-level sensitivity/PPV/NPV with Wilson 95% CIs; specificity/NPV noted as NE due to no IMI-S isolates, not estimable, NE).

| City | BMD‑R (n) | PCR+ | TP | FP | FN | TN | Sensitivity (95% CI) | Specificity (95% CI) | PPV (95% CI) | NPV (95% CI) |
| --- | --- | --- | --- | --- | --- | --- | --- | --- | --- | --- |
| Makkah | 35 | 35 | 35 | 0 | 0 | 0 | 100.0% (90.1–100) | NE* | 100.0% (90.1–100) | NE* |
| Riyadh | 36 | 33 | 33 | 0 | 3 | 0 | 91.7% (78.2–97.1) | NE* | 100.0% (89.6–100) | NE* |
| Combined | 71 | 68 | 68 | 0 | 3 | 0 | 95.8% (88.3–98.6) | NE* | 100.0% (94.7–100) | NE* |

NE* = Not estimable: no imipenem‑susceptible isolates in this cohort; specificity/NPV cannot be computed. Wilson 95% CIs are shown for proportions with non‑zero denominators. PPV is assumed to be 0 in these cohorts.

**Table S3.** Primers and PCR cycling conditions for Class B and Class D carbapenemase genes used in this study. Spiked-in positive control (ATCC **19606)** to check for inhibition. A positive result for spiked-in positive control confirms a true negative for the target gene. Added a known concentration of *E. coli* DNA to the original sample before extraction to serve as a positive extraction control.

| Class | Primer | Target gene | Primer sequence (5′→3′) | Amplicon (bp) | Cycling conditions | Reference |
| --- | --- | --- | --- | --- | --- | --- |
| Class B | IMP | *blaIMP* | F: GGAATAGAGTGGCTTAAYTCTC R: CCAAACYACTASGTTATCT | 188 | Initial denaturation at 94°C for 5 min; 30 cycles of 94°C for 40 s, 54°C for 1 min, 72°C for 2 min; final extension 72°C for 5 min. | Ellington et al., 2006 |
| Class B | *blaNDM* | *blaNDM* | F: CACCTCATGTTTGAATTCGCC R: CTCTGTCACATCGAAATCGC | 984 | Initial denaturation at 94°C for 5 min; 30 cycles of 94°C for 40 s, 54°C for 1 min; final extension 72°C for 5 min. | Poirel et al., 2010 |
| Class D | *blaOXA-23* | *blaOXA-23* | F: GATGTGTCATAGTATTCGTCGT R: TCACAACAACTAAAAGCACTGT | 1037 | Initial denaturation at 94°C for 5 min; 30 cycles of 94°C for 50 s, 55°C for 30 s, 72°C for 45 s; final extension 72°C for 2 min. | Jeon et al., 2005 |
| Class D | *blaOXA-51-Like* | *blaOXA-51-Like* | F: TAATGCTTTGATCGGCCTTG R: TGGATTGCACTTCATCTTGG | 353 | Initial denaturation at 94°C for 5 min; 30 cycles of 94°C for 50 s, 55°C for 30 s, 72°C for 45 s; final extension 72°C for 2 min. | Woodford et al., 2006 |

Notes: Sequences are 5′→3′. Degenerate bases follow IUPAC nomenclature—cycling conditions reported as initial denaturation; cycles (denaturation/annealing/extension); final extension.

**Table S4**. Predictive performance of PCR (*blaOXA-23*/*blaNDM*) versus BMD carbapenem resistance. City-stratified and overall TP/FP/FN/TN, PPV/NPV, sensitivity/specificity with Wilson 95% CIs. (Non-CR isolates were absent; NPV/specificity not defined).

| City | n | TP | FP | FN | TN | PPV % (95% CI) | NPV % (95% CI) | Sensitivity % (95% CI) | Specificity % (95% CI) |
| --- | --- | --- | --- | --- | --- | --- | --- | --- | --- |
| Makkah | 35 | 35 | 0 | 0 | 0 | 100.0% (90–100%) | NA (no non-CR isolates) | 100.0% (90–100%) | NA (no non-CR isolates) |
| Riyadh | 36 | 33 | 0 | 3 | 0 | 100.0% (90–100%) | NA (no non-CR isolates) | 91.7% (78–97%) | NA (no non-CR isolates) |
| Overall | 71 | 68 | 0 | 3 | 0 | 100.0% (95–100%) | NA (no non-CR isolates) | 95.8% (88–99%) | NA (no non-CR isolates) |

Notes: Reference = BMD carbapenem resistance (R to imipenem and/or meropenem). PPV = TP/(TP+FP); NPV = TN/(TN+FN); Sensitivity = TP/(TP+FN); Specificity = TN/(TN+FP). 95% CIs are Wilson. Non-CR isolates were absent in both cities; therefore, NPV and specificity are not defined.


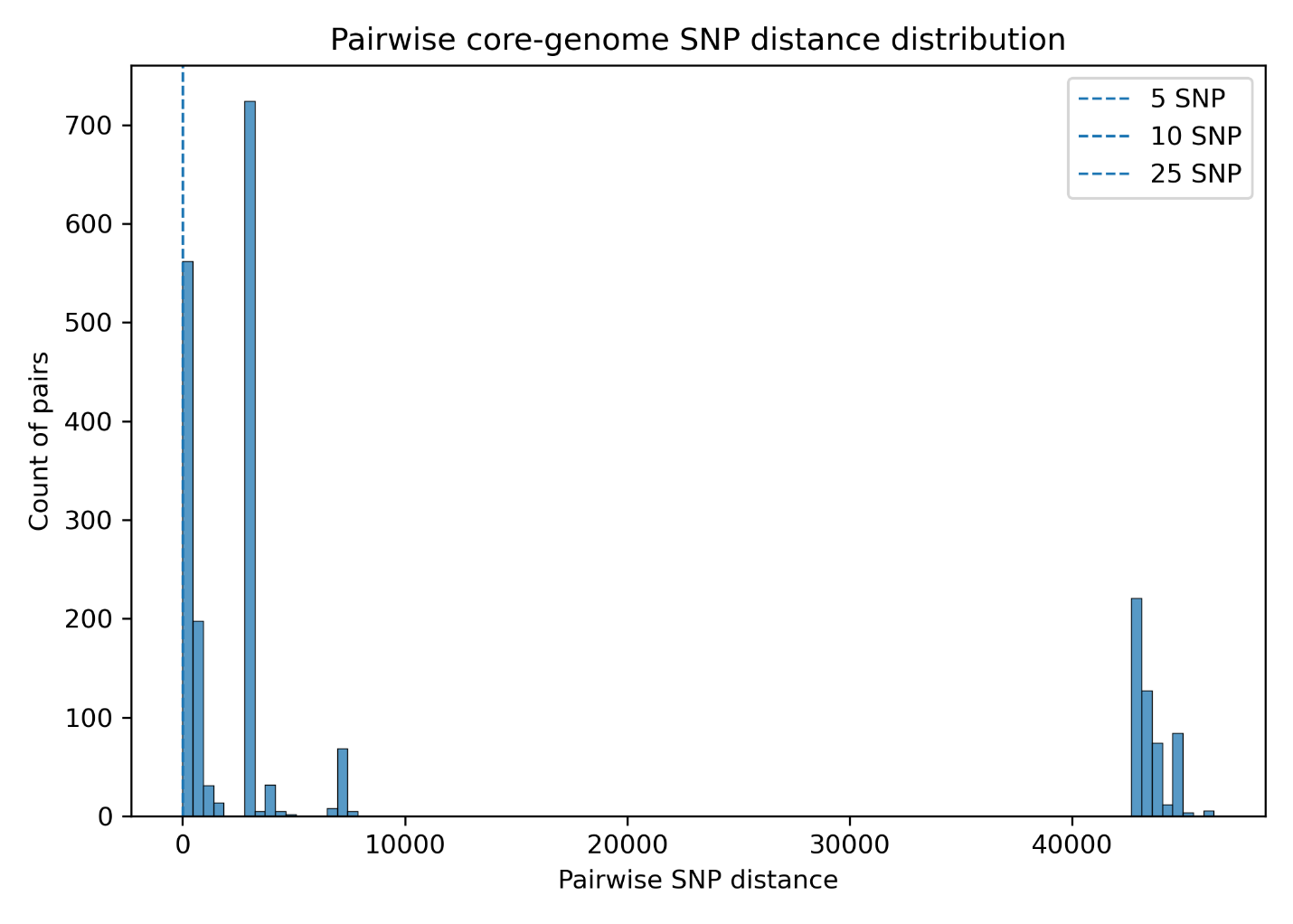
**Figure S5.** Pairwise core-genome SNP distance distribution. Histogram of pairwise SNP distances from the recombination-filtered core alignment. Vertical dashed lines mark 5, 10, and 25 SNPs (the thresholds used in sensitivity checks). Most isolate pairs fall well above 10 SNPs, while a smaller group lies ≤10 SNPs, which we treat as putative recent transmission links. This distribution supports our operational ≤10-SNP cluster definition used in Figure 4 and in the cluster analyses (supplementary Figure S6 and supplementary Table S5), showing that “close pairs” (≤10 SNPs) are a minority and therefore likely meaningful for IPC triage rather than background noise.


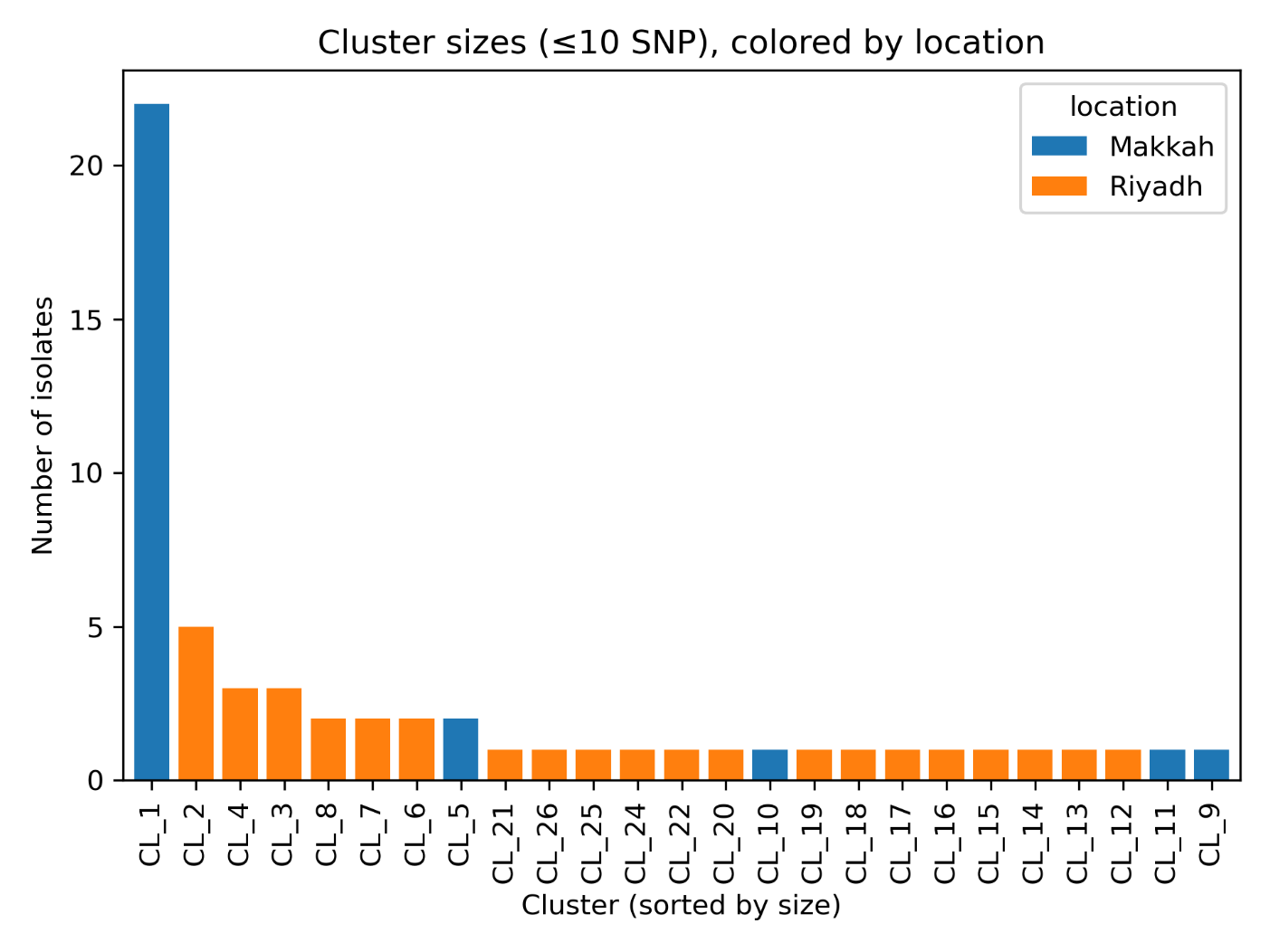


### **Figure S6.** Cluster membership and cross-city mixing (≤10-SNP threshold). Stacked bars show cluster sizes and city composition (Makkah/Riyadh). Most clusters are small and single-city; mixed-city clusters: 0%. Permutation test for excess mixing (5,000 permutations): p = 1.0. See Supplementary Table S5 for per-cluster counts.

**Table S5.** Cluster membership and city composition.

| Cluster ID | Makkah (n) | Riyadh (n) | Size | Mixed |
| --- | --- | --- | --- | --- |
| CL_1 | 22 | 0 | 22 | No |
| CL_2 | 0 | 5 | 5 | No |
| CL_3 | 0 | 3 | 3 | No |
| CL_4 | 0 | 3 | 3 | No |
| CL_5 | 2 | 0 | 2 | No |
| CL_6 | 0 | 2 | 2 | No |
| CL_7 | 0 | 2 | 2 | No |
| CL_8 | 0 | 2 | 2 | No |

Notes: Mixed = any cluster containing isolates from both cities; here, all clusters are single-city (0% mixed).


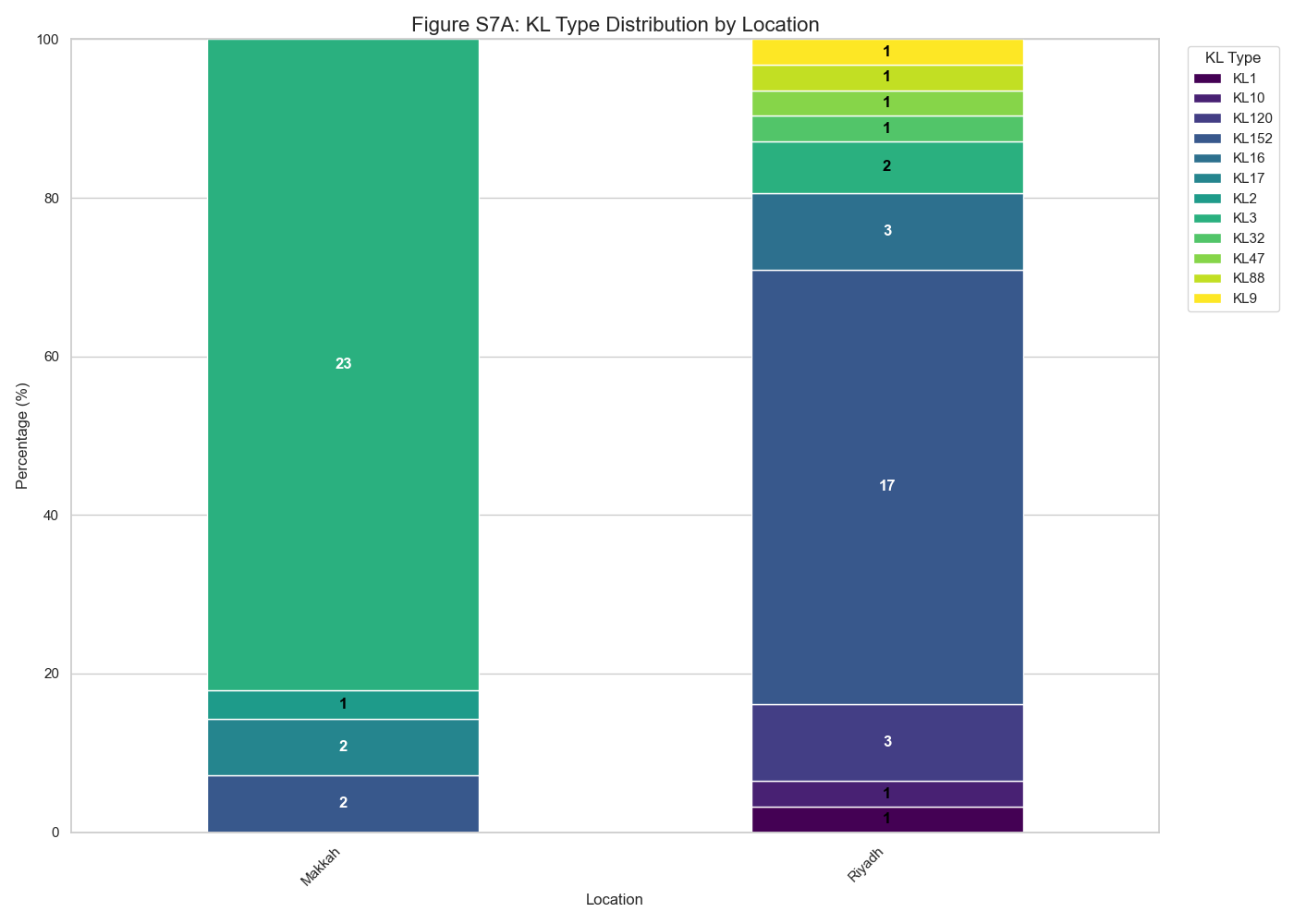


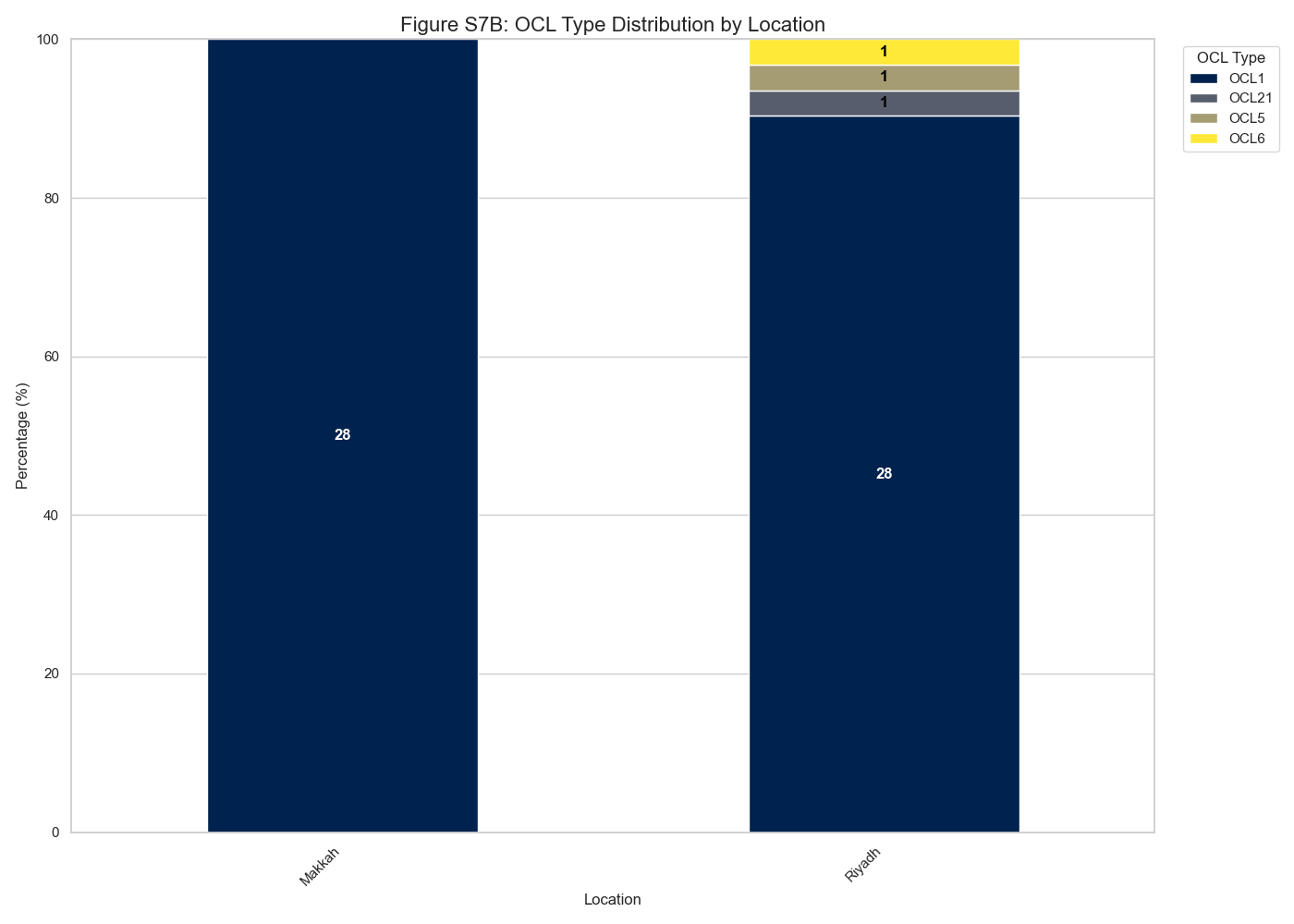
**Figure S7.** KL and OCL distributions (Kaptive, confidence ≥ good). Distribution of **capsule (KL)** *and* **LOS outer-core (OCL)** locus types called by Kaptive (only **“good”** or higher confidence shown; lower confidence grouped as “other”). **(A)** Stacked bar plots of KL types among *A. baumannii* isolates from Riyadh and Makkah. **(B)** Stacked bar plots of OCL types by city. KL/OCL types are **diverse across the two cities** within the **GC2/ST2-dominant** background observed in the phylogeny, suggesting that **no single capsule or LOS type** is driving city-specific patterns. This supports our conclusion that the **population structure (GC2/ST2) and local transmission** explain most of the signal, rather than a KL/OCL-specific expansion.


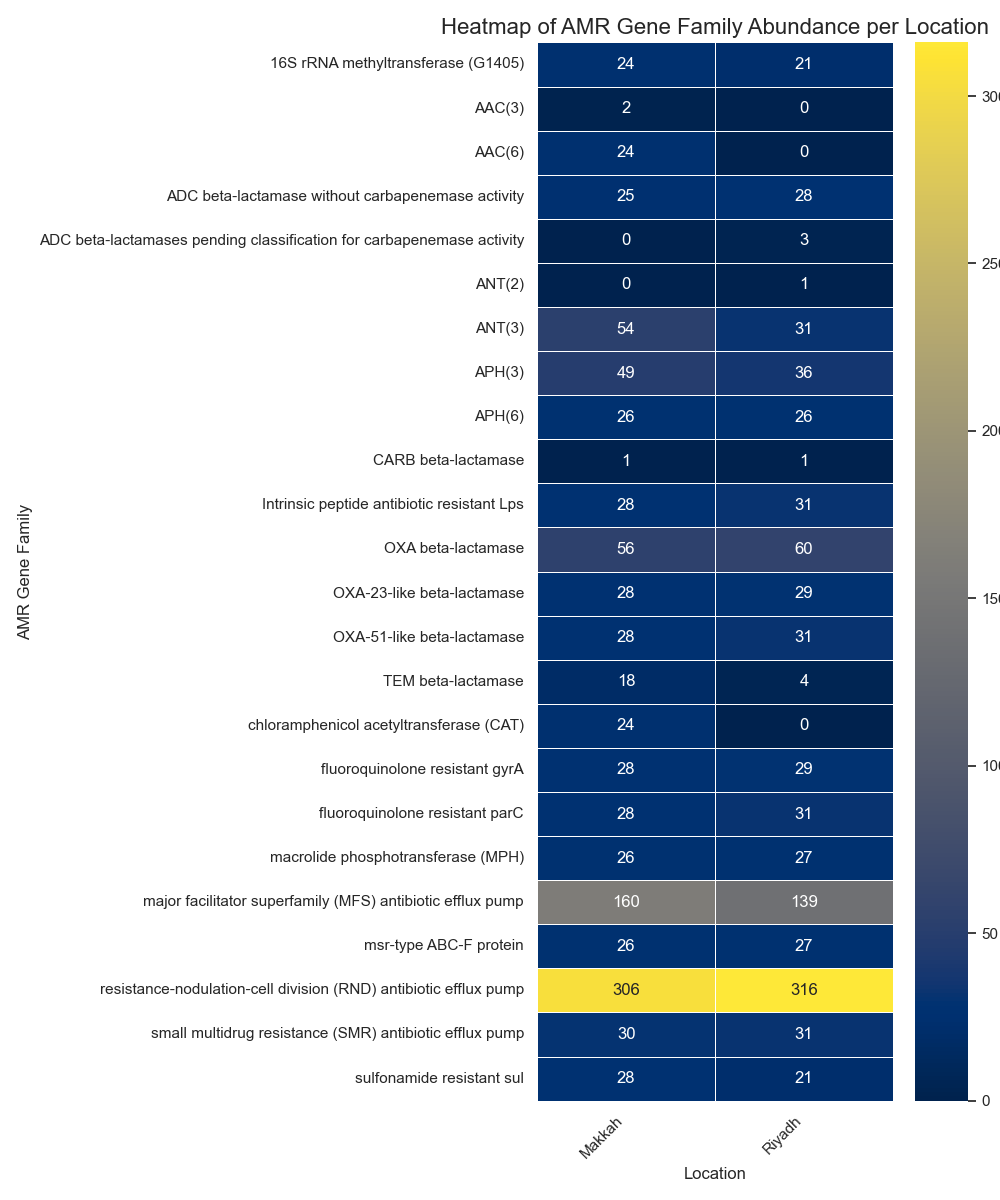
**Figure S8.** AMR gene family abundance per city (AMRFinderPlus).

Heatmap shows total counts of detected AMR gene families in Makkah and Riyadh across major mechanism classes (e.g., carbapenemases, β-lactamases, aminoglycoside-modifying enzymes, quinolone resistance determinants, and multidrug efflux systems). Profiles are broadly similar across cities and align with the phenotypic MDR burden reported in Table 1.


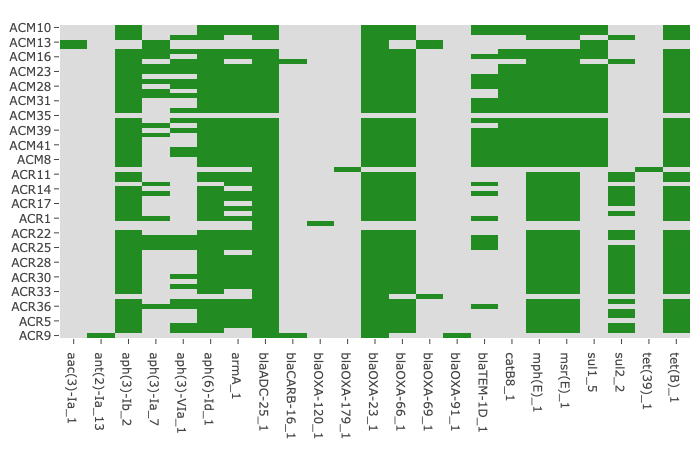


**Figure S9**. Resistome presence/absence heatmap (AMRFinderPlus). Rows are AMR genes grouped by class; columns are isolates (Makkah then Riyadh). Dark cells = gene detected. The shared backbone blaOXA-23 (±blaNDM) with frequent armA/AMEs, msrE/mphE, tet(B)/tetR, sul1/qacEΔ1, and ade-family efflux systems accounts for the MDR phenotype observed in BMD and supports the high PPV of focused carbapenemase PCR in this cohort.

**
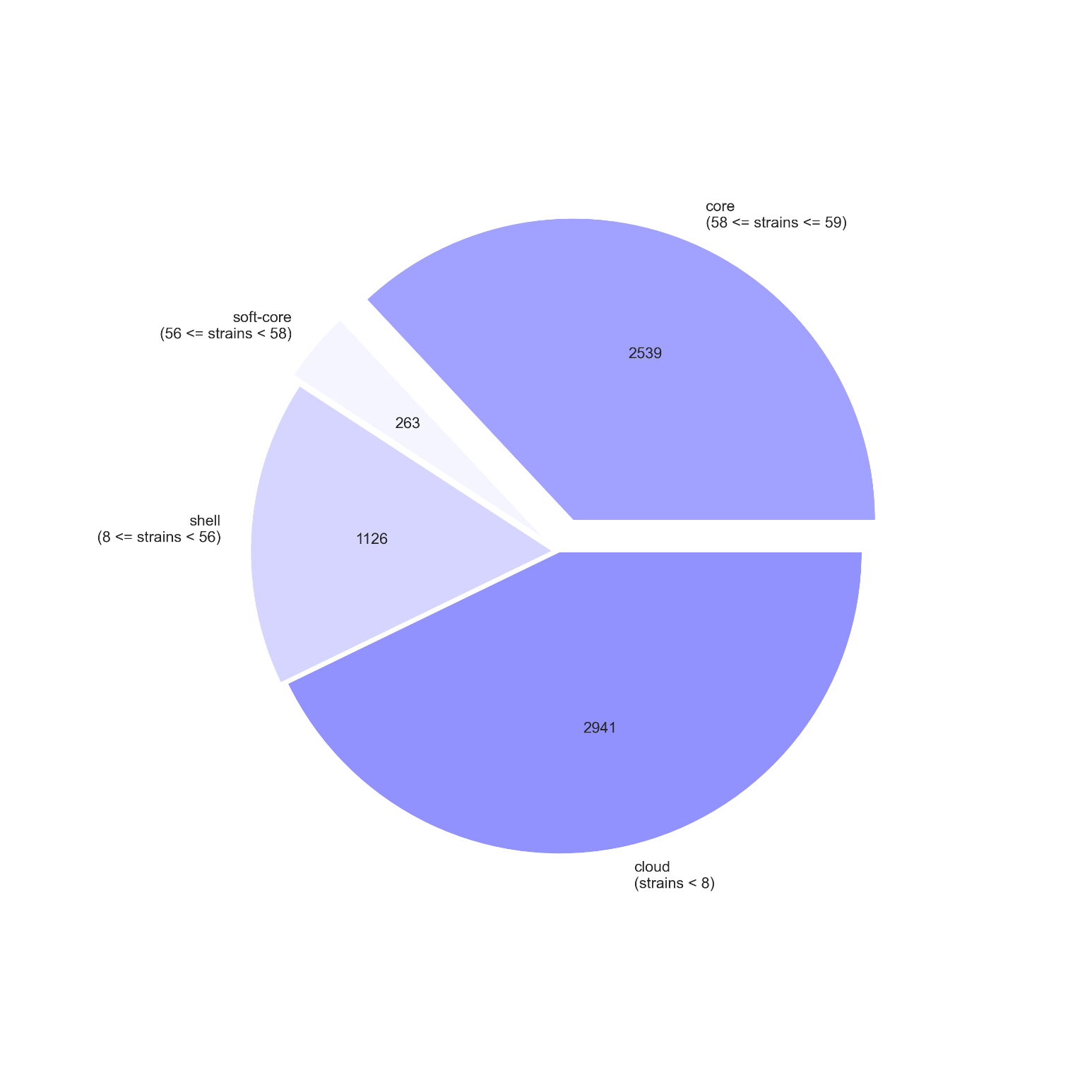
**

**Figure S10.** Pangenome composition of 59 *A. baumannii* genomes. Pie chart showing the number and proportion of gene clusters in each category (total = 6,869): core (present in ≥58/59 isolates) = 2,539 (37%); soft-core (56–57/59) = 263 (3.8%); shell (8–55/59) = 1,126 (16%); cloud (<8/59) = 2,941 (43%). Categories were defined from Roary output at 95% protein identity. A large cloud fraction reflects an open accessory genome typical of *A. baumannii* and is consistent with the GC2/ST2-dominant but polyclonal structure observed in the core-genome tree.

**Abbreviations**: BMD, broth microdilution; VME, Very Major Error; ME, Major Error; MIC, minimum inhibitory concentration; CLSI, Clinical & Laboratory Standards Institute; IMI, imipenem; MEM, meropenem; CAZ, ceftazidime; CIP, ciprofloxacin; WGS, whole-genome sequencing; MLST, multilocus sequence typing; KL/OCL, capsular/lipooligosaccharide loci. CRAB: carbapenem-resistant *A. baumannii*
IPC: infection prevention and control PPV / NPV: positive predictive value / negative predictive value IC2 / GC2: International Clone 2 / Global Clone 2 LOS: lipooligosaccharide Δ%R: difference in percent resistant calls between VITEK II and BMD NE: not estimable.
