# Supplementary material for: Phenotypic, PCR, and whole-genome sequencing characterization of carbapenem-resistant Acinetobacter baumannii in Makkah and Riyadh, Saudi Arabia: AST discordance and local genomic micro-clusters
Source: Front Microbiol. 2026 Jun 22;17:1845444. doi: 10.3389/fmicb.2026.1845444 (PMC13333605; doi:10.3389/fmicb.2026.1845444)
Supplement: Supplementary file 2 [file Table_2.DOCX]

**Supplementary Materials**

**Table S0. An anonymized isolate metadata summary and fields were provided for this study.**

| **Field** | **Description** |
| --- | --- |
| Isolate code | An anonymized isolate identifier used throughout the phenotype, PCR, and WGS analyses. |
| City | Sampling city: Makkah or Riyadh. |
| Specimen/source category | Routine clinical specimen category, including sputum, wound swab, tracheal aspirate, bronchial wash, blood, tissue, drain, or urine. |
| BMD phenotype | Reference broth microdilution categorical interpretation for imipenem, meropenem, ceftazidime, and ciprofloxacin. |
| PCR result | Presence/absence of blaOXA-51-like, blaOXA-23, and blaNDM by targeted PCR. |
| WGS QC status | Sequencing library retained or excluded from comparative genomic analysis according to read-assignment, assembly, and ANI quality filters. |
| Cluster ID | WGS micro-cluster assignment at the ≤10 core-SNP threshold for genomes passing WGS QC. |

Table S1. BMD susceptibility summary by drug and city. MIC, minimum inhibitory concentration; IQR, interquartile range; IMI, imipenem; MEM, meropenem; CAZ, ceftazidime; CIP, ciprofloxacin.

| **Drug** | **City** | **n** | **S** | **I** | **R** | **R %** | **95% CI** | **Median MIC [IQR]** | **Fisher’s p (city vs city)** |
| --- | --- | --- | --- | --- | --- | --- | --- | --- | --- |
| IMI | Makkah | 35 | 0 | 0 | 35 | 100.0 | 90.1-100.0 | 1024 [1024-1024] | 1.000 |
| IMI | Riyadh | 36 | 0 | 0 | 36 | 100.0 | 90.4-100.0 | 1024 [1024-1024] |  |
| MEM | Makkah | 35 | 0 | 0 | 35 | 100.0 | 90.1-100.0 | 128 [128-192] | 1.000 |
| MEM | Riyadh | 36 | 1 | 0 | 35 | 97.2 | 85.8-99.5 | 128 [64-256] |  |
| CAZ | Makkah | 35 | 0 | 0 | 35 | 100.0 | 90.1-100.0 | 512 [256-512] | 0.239 |
| CAZ | Riyadh | 36 | 3 | 0 | 33 | 91.7 | 78.2-97.1 | 256 [128-320] |  |
| CIP | Makkah | 35 | 0 | 0 | 35 | 100.0 | 90.1-100.0 | 128 [128-128] | 0.493 |
| CIP | Riyadh | 36 | 1 | 1 | 34 | 94.4 | 81.9-98.5 | 16 [16-64] |  |

BMD, Broth Microdilution; S, Susceptible; I, Intermediate; R, Resistant; MIC, Minimum Inhibitory 342 Concentration; IQR, Interquartile Range. Statistical Analysis: Resistance percentages are presented with their 343 95% Wilson Confidence Intervals. The two-sided Fisher p-value compares the R% between Makkah and 344 Riyadh for the given drug.

Table S2. VITEK II categorical results by city and selected antimicrobial agent. S, susceptible; I, intermediate; R, resistant.

| **City** | **Drug** | **S** | **I** | **R** |
| --- | --- | --- | --- | --- |
| Makkah | Imipenem | 0 | 0 | 35 |
| Makkah | Meropenem | 0 | 0 | 35 |
| Makkah | Ciprofloxacin | 0 | 0 | 35 |
| Makkah | Ceftazidime | 2 | 0 | 33 |
| Makkah | Cefepime | 0 | 0 | 35 |
| Makkah | Gentamicin | 2 | 0 | 33 |
| Makkah | Tigecycline | 13 | 20 | 2 |
| Makkah | Trimethoprim/Sulfamethoxazole | 0 | 0 | 35 |
| Riyadh | Imipenem | 2 | 0 | 32 |
| Riyadh | Meropenem | 2 | 1 | 31 |
| Riyadh | Ciprofloxacin | 2 | 0 | 32 |
| Riyadh | Ceftazidime | 2 | 1 | 31 |
| Riyadh | Cefepime | 2 | 0 | 32 |
| Riyadh | Gentamicin | 11 | 0 | 23 |
| Riyadh | Tigecycline | 30 | 1 | 3 |
| Riyadh | Trimethoprim/Sulfamethoxazole | 11 | 0 | 23 |

**Table S3. Minimum inhibitory concentration (MIC) determination in A. baumannii clinical isolates from the Makkah region.**

| **A. baumannii** | **Ciprofloxacin R ≥ 4 µg/ml** | **Ceftazidime R ≥ 32µg/ml** | **Meropenem R≥ 8 µg/ml** | **Imipenem R ≥ 8 µg/ml** |
| --- | --- | --- | --- | --- |
| 1 | 8 | ≥1024 | 128 | ≥1024 |
| 2 | 128 | 1024 | 256 | ≥1024 |
| 3 | 256 | 128 | 128 | ≥1024 |
| 8 | 128 | 256 | 128 | ≥1024 |
| 9 | 128 | 256 | 256 | ≥1024 |
| 10 | 128 | 512 | 64 | ≥1024 |
| 11 | 128 | 512 | 128 | ≥1024 |
| 12 | 64 | 128 | 256 | ≥1024 |
| 13 | 8 | 256 | 32 | ≥1024 |
| 14 | 8 | 128 | 128 | ≥1024 |
| 15 | 128 | 512 | 128 | ≥1024 |
| 16 | 128 | 1204 | 64 | ≥1024 |
| 17 | 128 | 512 | 128 | ≥1024 |
| 18 | 128 | 512 | 128 | ≥1024 |
| 19 | 128 | 512 | 128 | ≥1024 |
| 21 | 128 | 1204 | 128 | ≥1024 |
| 22 | 128 | 256 | 256 | ≥1024 |
| 23 | 128 | 512 | 128 | ≥1024 |
| 26 | 128 | 512 | 256 | ≥1024 |
| 27 | 128 | 256 | 128 | ≥1024 |
| 28 | 128 | 256 | 128 | ≥1024 |
| 29 | ≥1024 | 256 | 128 | ≥1024 |
| 30 | 128 | 256 | 128 | ≥1024 |
| 31 | 128 | 256 | 256 | ≥1024 |
| 32 | 64 | ≥1024 | 128 | ≥1024 |
| 33 | 128 | 512 | 128 | ≥1024 |
| 34 | 128 | 512 | 128 | ≥1024 |
| 35 | 128 | 512 | 64 | ≥1024 |
| 36 | 128 | 512 | 64 | ≥1024 |
| 37 | 128 | 512 | 128 | ≥1024 |
| 39 | 128 | 32 | ≥1024 | ≥1024 |
| 40 | 64 | 512 | ≥1024 | ≥1024 |
| 41 | 64 | 512 | ≥1024 | ≥1024 |
| 42 | 128 | 512 | 32 | ≥1024 |
| 43 | 64 | 512 | 128 | ≥1024 |
| Resistance ratio | 100% R | 100% R | 100% R | 100% R |

**Table S4. Minimum inhibitory concentration (MIC) determination in A. baumannii clinical isolates from the Riyadh region.**

| **A. baumannii** | **Ciprofloxacin  R ≥ 4 µg/ml** | **Ceftazidime  R ≥ 32µg/ml** | **Meropenem  R ≥ 8 µg/ml** | **Imipenem   R ≥ 8 µg/ml** |
| --- | --- | --- | --- | --- |
| 1 | 128 | 512 | 64 | ≥1024 |
| 2 | 8 | 256 | 64 | ≥1024 |
| 3 | 8 | 128 | 64 | ≥1024 |
| 4 | 8 | 128 | 32 | ≥1024 |
| 5 | 128 | 1024 | 32 | ≥1024 |
| 6 | 64 | 32 | 64 | ≥1024 |
| 7 | 16 | 256 | 64 | ≥1024 |
| 8 | 16 | 64 | 16 | ≥1024 |
| 9 | 64 | 8 | 64 | ≥1024 |
| 10 | 8 | 64 | 8 | ≥1024 |
| 11 | 16 | 128 | 256 | ≥1024 |
| 12 | 32 | 128 | 256 | ≥1024 |
| 13 | 128 | 512 | 256 | ≥1024 |
| 14 | 16 | 128 | 128 | ≥1024 |
| 15 | 256 | 128 | 256 | ≥1024 |
| 16 | 16 | 256 | 256 | ≥1024 |
| 17 | 16 | 128 | 128 | ≥1024 |
| 18 | 64 | 512 | 256 | ≥1024 |
| 19 | 16 | 128 | 128 | ≥1024 |
| 20 | 32 | 512 | 32 | 8 |
| 21 | 0.5 | 8 | 0.5 | 128 |
| 22 | 128 | 512 | 256 | ≥1024 |
| 23 | 16 | 256 | 256 | ≥1024 |
| 24 | 64 | 512 | 128 | ≥1024 |
| 25 | 32 | 512 | 256 | ≥1024 |
| 26 | 1024 | 128 | 64 | ≥1024 |
| 27 | 32 | 128 | 128 | ≥1024 |
| 28 | 32 | 128 | 128 | ≥1024 |
| 29 | 8 | 256 | 128 | ≥1024 |
| 30 | 16 | 256 | 128 | ≥1024 |
| 31 | 32 | 256 | 512 | ≥1024 |
| 32 | 16 | 256 | 128 | ≥1024 |
| 33 | 16 | 256 | 128 | ≥1024 |
| 34 | 2 | 8 | 64 | ≥1024 |
| 35 | 16 | 256 | 256 | ≥1024 |
| 36 | 64 | 1024 | 256 | ≥1024 |
| Resistance ratio | R 94% (34/36) S 3% (1/36) I 3% (1/36) | R 91.50% (33/36) S 8.5% (3/36) | R 97% (35/36) S 3% (1/36) | R 100% (36/36) |

**Table S5. Diagnostic performance of composite carbapenemase PCR (blaOXA-23 OR blaNDM) versus imipenem BMD.**

| **City** | **BMD‑R (n)** | **PCR+** | **TP** | **FP** | **FN** | **TN** | **Sensitivity (95% CI)** | **Specificity (95% CI)** | **PPV (95% CI)** | **NPV (95% CI)** |
| --- | --- | --- | --- | --- | --- | --- | --- | --- | --- | --- |
| Makkah | 35 | 35 | 35 | 0 | 0 | 0 | 100.0% (90.1–100) | NE* | 100.0% (90.1–100) | NE* |
| Riyadh | 36 | 33 | 33 | 0 | 3 | 0 | 91.7% (78.2–97.1) | NE* | 100.0% (89.6–100) | NE* |
| Combined | 71 | 68 | 68 | 0 | 3 | 0 | 95.8% (88.3–98.6) | NE* | 100.0% (94.7–100) | NE* |

**Table S6. Primers and PCR cycling conditions for Class B and Class D carbapenemase genes used in this study.**

| **Class** | **Primer** | **Target gene** | **Primer sequence (5′→3′)** | **Amplicon (bp)** | **Cycling conditions** | **Reference** |
| --- | --- | --- | --- | --- | --- | --- |
| Class B | IMP | blaIMP | F: GGAATAGAGTGGCTTAAYTCTC R: CCAAACYACTASGTTATCT | 188 | Initial denaturation at 94°C for 5 min; 30 cycles of 94°C for 40 s, 54°C for 1 min, 72°C for 2 min; final extension 72°C for 5 min. | Ellington et al., 2006 |
| Class B | blaNDM | blaNDM | F: CACCTCATGTTTGAATTCGCC R: CTCTGTCACATCGAAATCGC | 984 | Initial denaturation at 94°C for 5 min; 30 cycles of 94°C for 40 s, 54°C for 1 min; final extension 72°C for 5 min. | Poirel et al., 2010 |
| Class D | blaOXA-23 | blaOXA-23 | F: GATGTGTCATAGTATTCGTCGT R: TCACAACAACTAAAAGCACTGT | 1037 | Initial denaturation at 94°C for 5 min; 30 cycles of 94°C for 50 s, 55°C for 30 s, 72°C for 45 s; final extension 72°C for 2 min. | Jeon et al., 2005 |
| Class D | blaOXA-51-Like | blaOXA-51-Like | F: TAATGCTTTGATCGGCCTTG R: TGGATTGCACTTCATCTTGG | 353 | Initial denaturation at 94°C for 5 min; 30 cycles of 94°C for 50 s, 55°C for 30 s, 72°C for 45 s; final extension 72°C for 2 min. | Woodford et al., 2006 |

**Figure S1. Pairwise core-genome SNP distance distribution. Histogram of pairwise SNP distances from the recombination-filtered core alignment. Vertical dashed lines mark 5, 10, and 25 SNPs.**


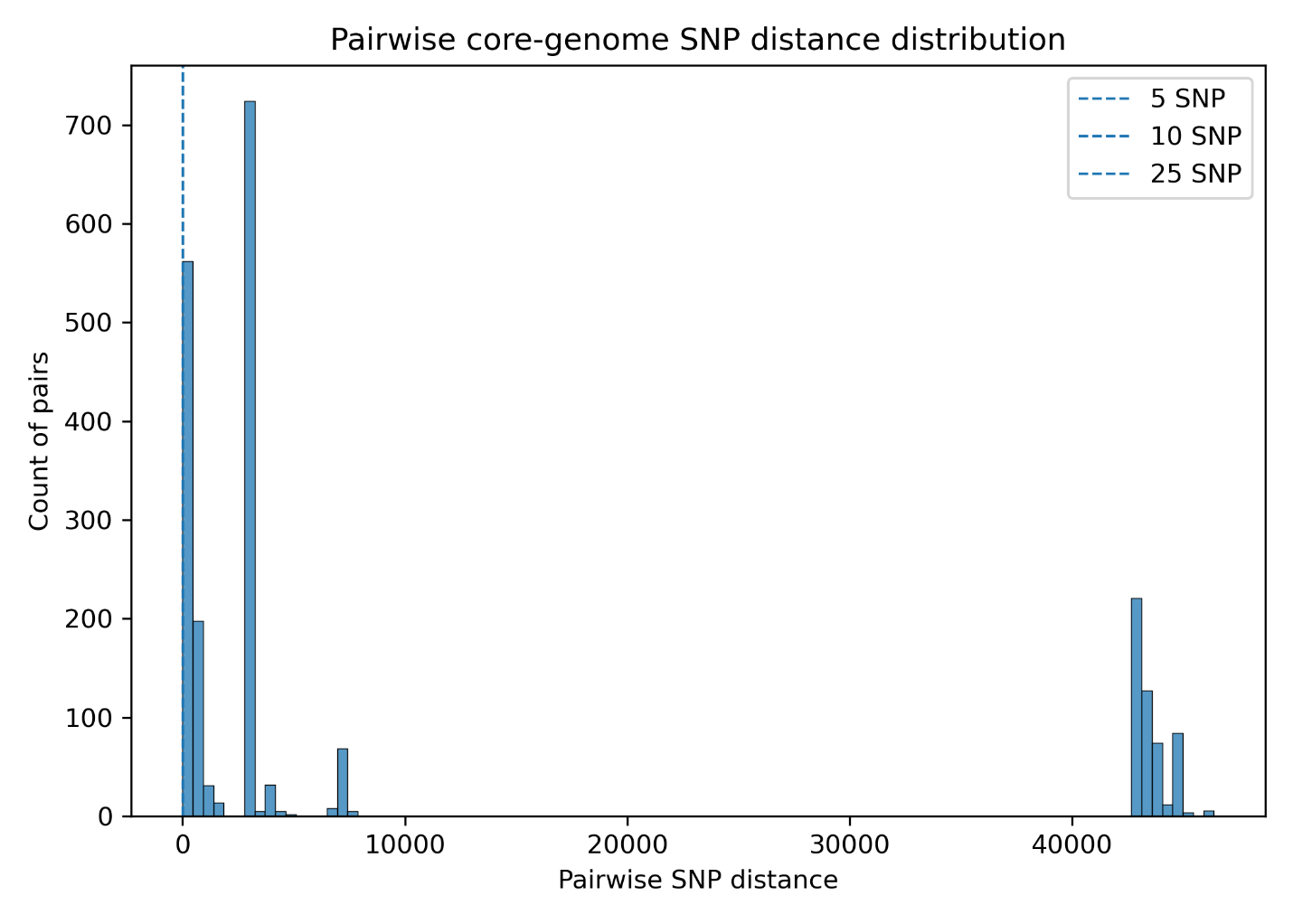


Pairwise core-genome SNP distance distribution. Histogram of pairwise SNP distances from the recombination-filtered core alignment. Vertical dashed lines mark 5, 10, and 25 SNPs (the thresholds used in sensitivity checks). Most isolate pairs fall well above 10 SNPs, while a smaller group lies ≤10 SNPs, which we treat as putative recent transmission links. This distribution supports our operational ≤10-SNP cluster definition used in Figure 4 and in the cluster analyses (supplementary Figure S6 and supplementary Table S5), showing that “close pairs” (≤10 SNPs) are a minority and therefore likely meaningful for IPC triage rather than background noise.

**Figure S2. Cluster membership and cross-city mixing at the ≤10-SNP threshold. Stacked bars show cluster sizes and city composition.**


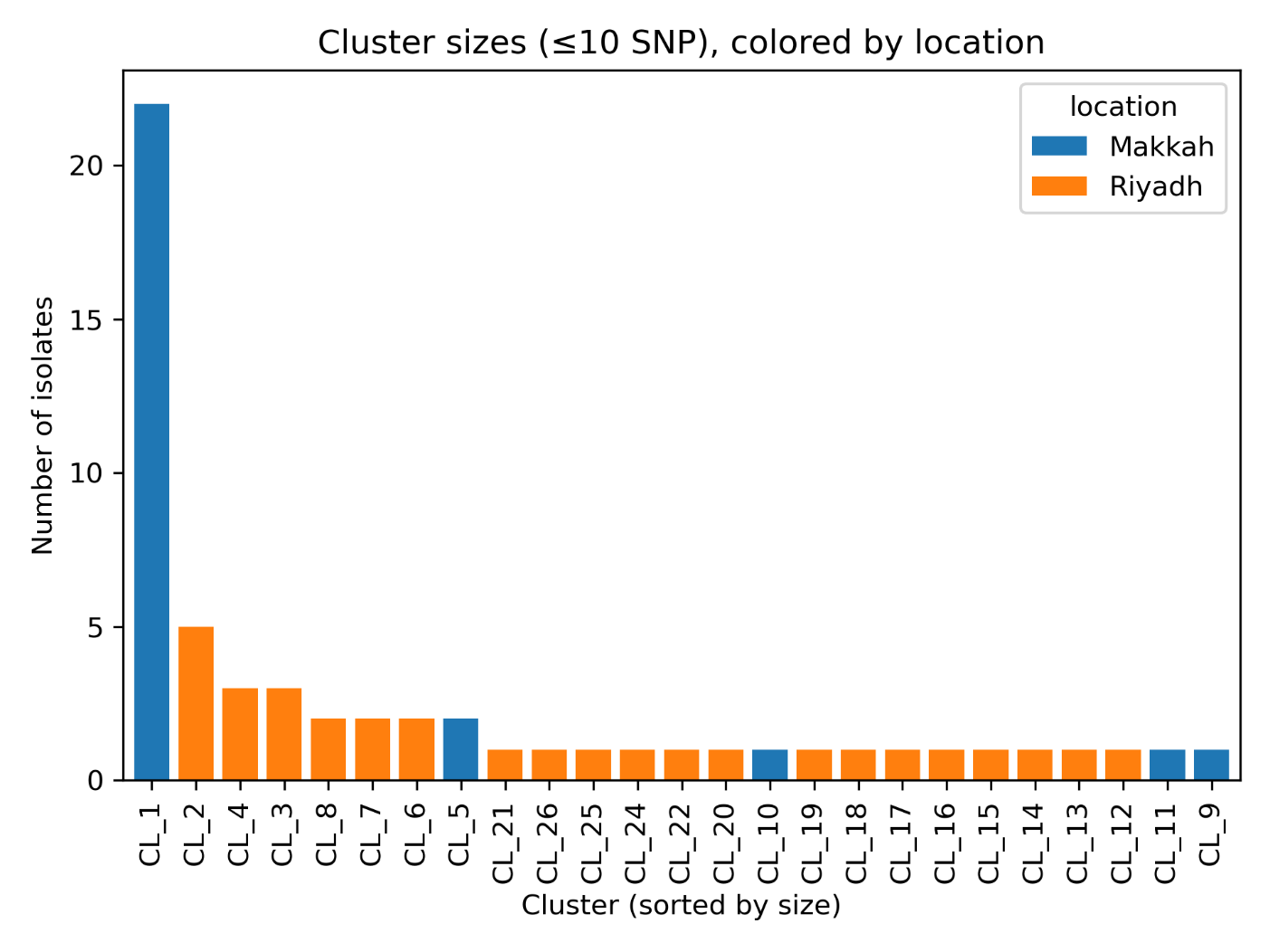


Cluster membership and cross-city mixing (≤10-SNP threshold). Stacked bars show cluster sizes and city composition (Makkah/Riyadh). Most clusters are small and single-city; mixed-city clusters: 0%. Permutation test for excess mixing (5,000 permutations): p = 1.0. See Supplementary Table S5 for per-cluster counts.

**Figure S3A. KL type distribution by location (Kaptive, confidence ≥ good).**


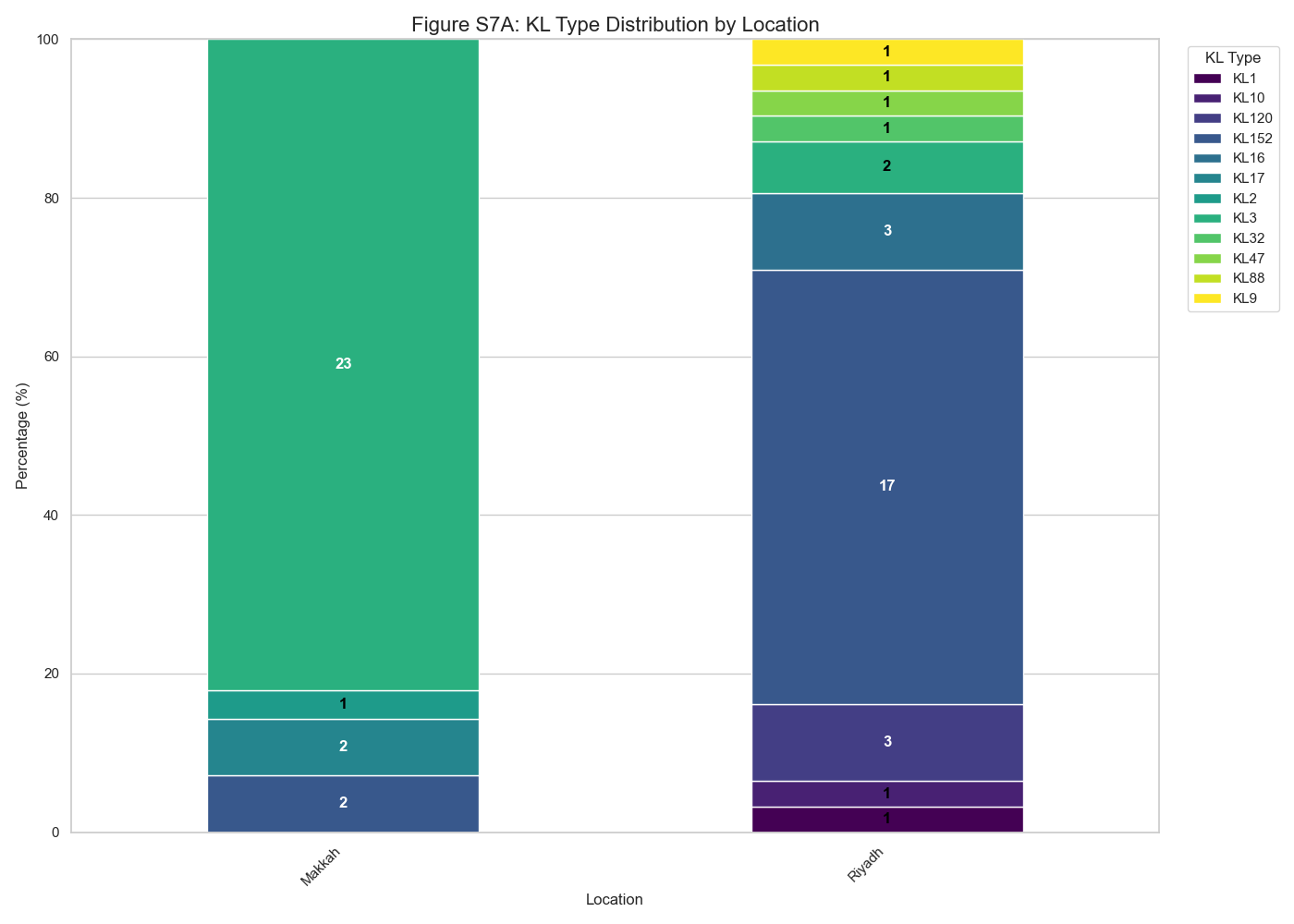


**Figure S3B. OCL type distribution by location (Kaptive, confidence ≥ good).**


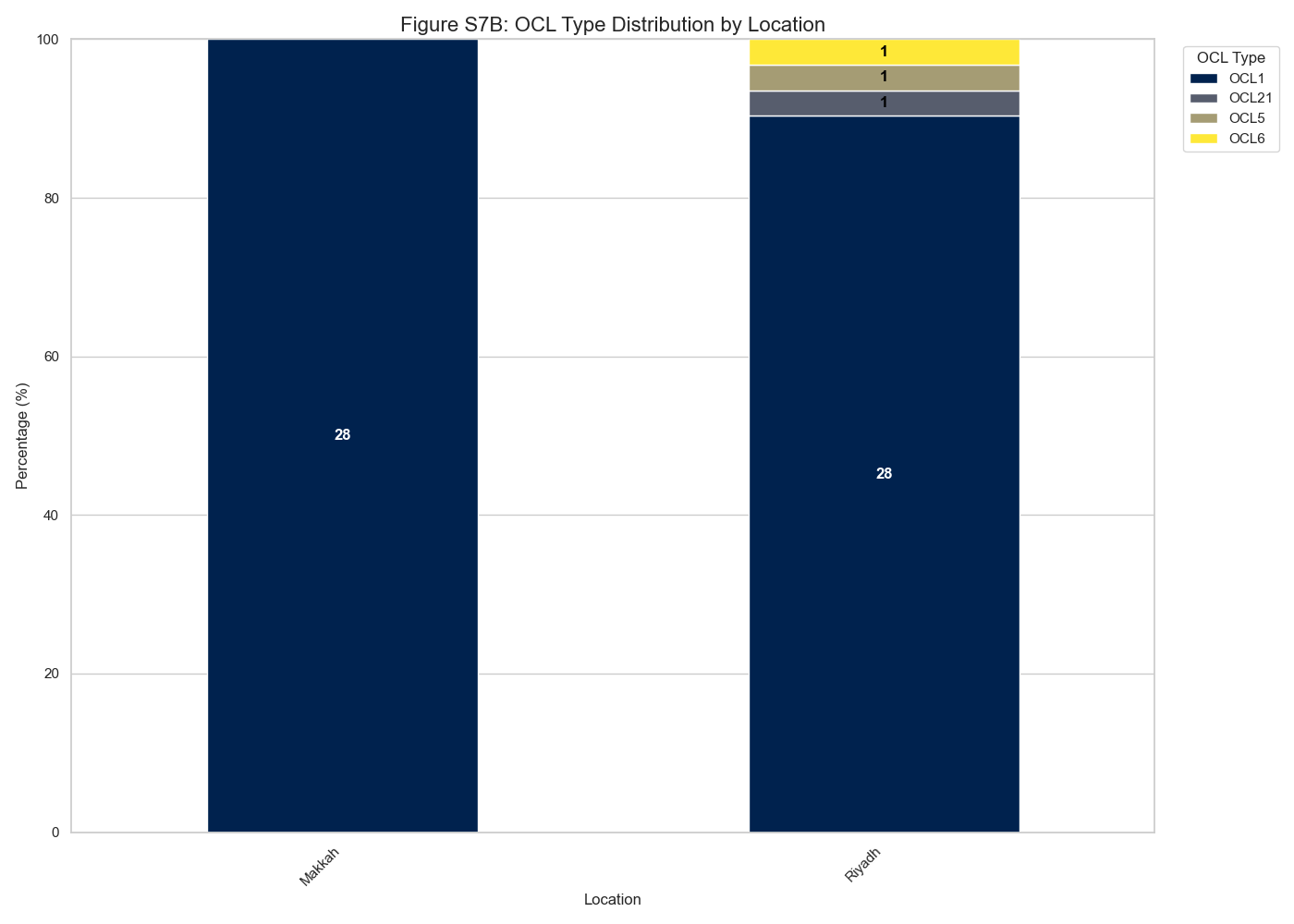


KL and OCL distributions (Kaptive, confidence ≥ good). Distribution of capsule (KL) and LOS outer-core (OCL) locus types called by Kaptive (only “good” or higher confidence shown; lower confidence grouped as “other”). (A) Stacked bar plots of KL types among *A. baumannii* isolates from Riyadh and Makkah. (B) Stacked bar plots of OCL types by city. KL/OCL types are diverse across the two cities within the GC2/ST2-dominant background observed in the phylogeny, suggesting that no single capsule or LOS type is driving city-specific patterns. This supports our conclusion that the population structure (GC2/ST2) and local transmission explain most of the signal, rather than a KL/OCL-specific expansion.

**Figure S4. AMR gene family abundance per city (AMRFinderPlus). The heatmap shows the total counts of detected AMR gene families in Makkah and Riyadh.**


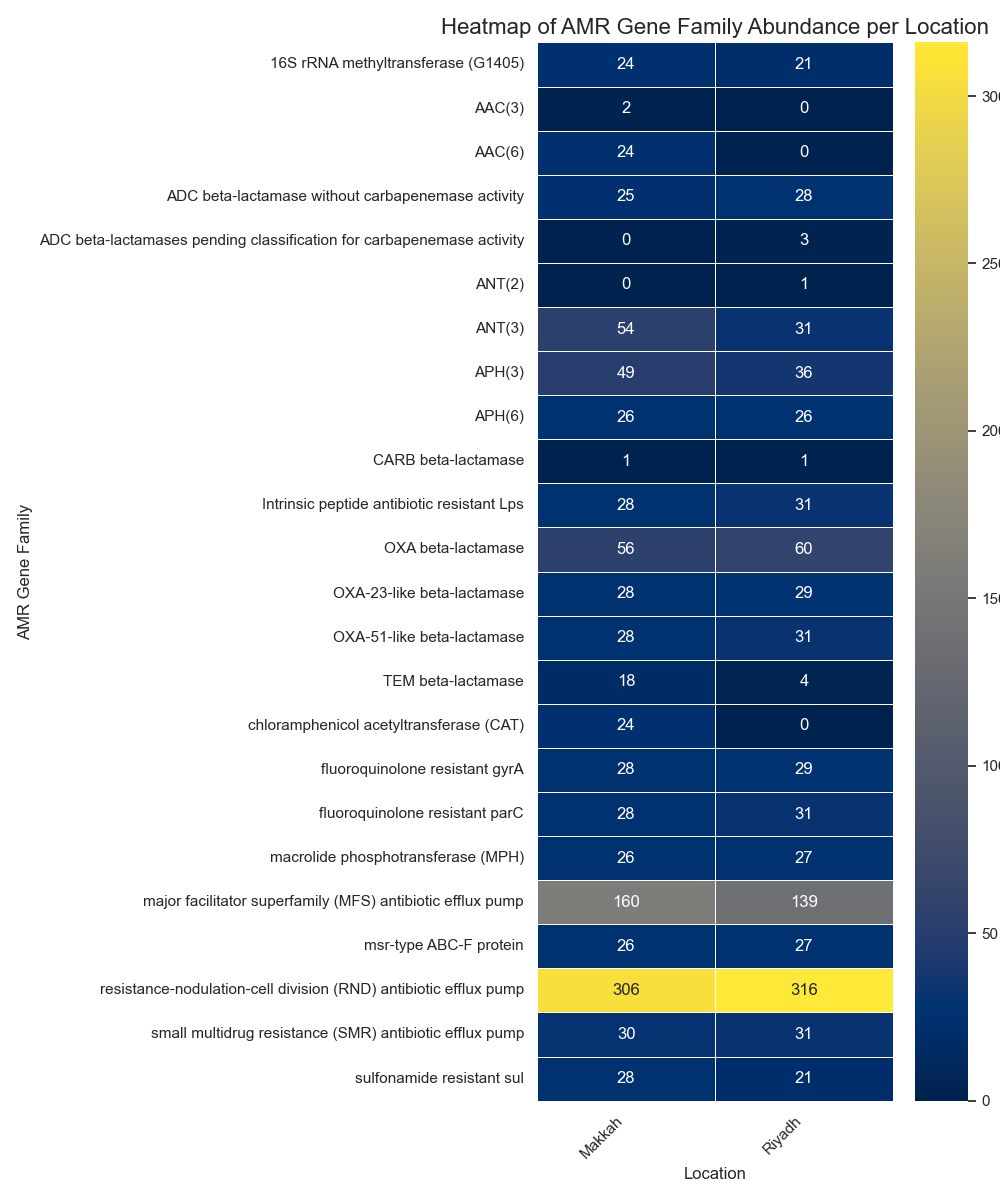


Heatmap shows total counts of detected AMR gene families in Makkah and Riyadh across major mechanism classes (e.g., carbapenemases, β-lactamases, aminoglycoside-modifying enzymes, quinolone resistance determinants, and multidrug efflux systems).

**Figure S5. Resistome presence/absence heatmap (AMRFinderPlus). Rows are AMR genes grouped by class; columns are isolates.**


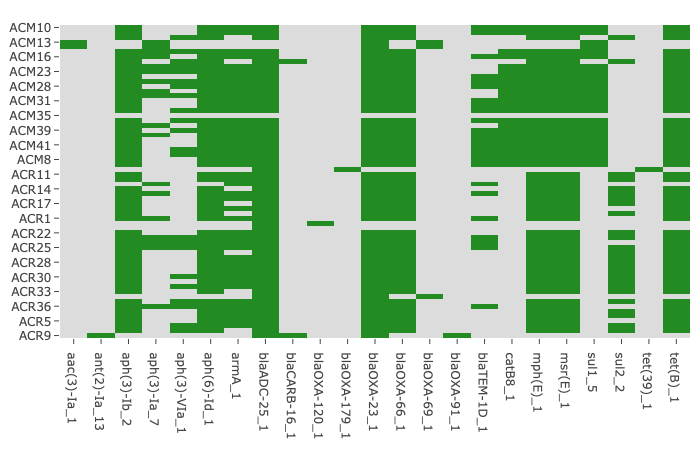


Resistome presence/absence heatmap (AMRFinderPlus). Rows are AMR genes grouped by class; columns are isolates (Makkah then Riyadh). Dark cells = gene detected. The shared backbone *blaOXA-23* (±*blaNDM*) with frequent *armA/AMEs, msrE/mphE, tet(B)/tetR*, *sul1/qacEΔ1*, and ade-family efflux systems accounts for the MDR phenotype observed in BMD and supports the high PPV of focused carbapenemase PCR in this cohort.

**Figure S6. Pangenome composition of 59 A. baumannii genomes. Pie chart showing the number and proportion of gene clusters in each category.**


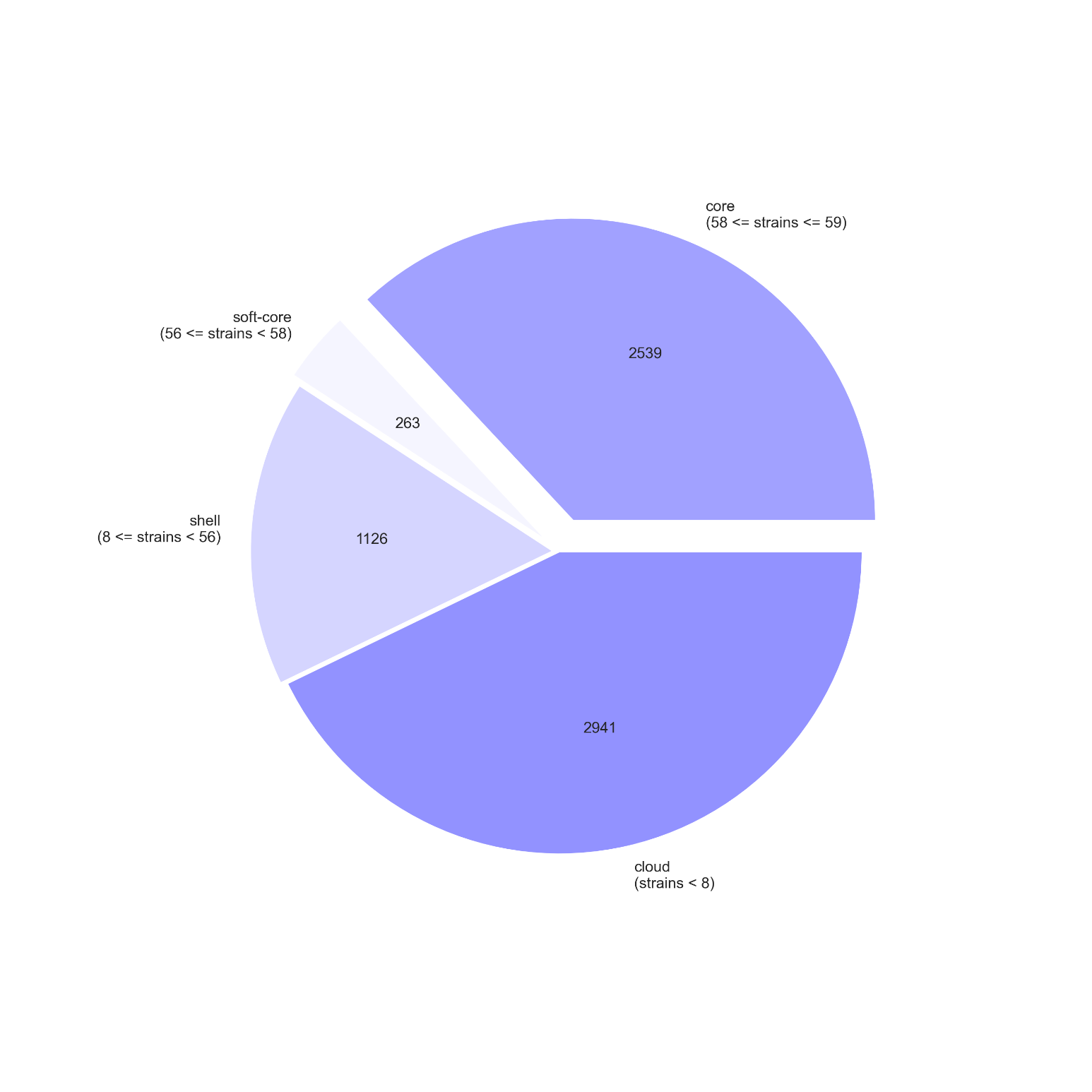


Abbreviations: BMD, broth microdilution; VME, very major error; ME, major error; MIC, minimum inhibitory concentration; CLSI, Clinical & Laboratory Standards Institute; IMI, imipenem; MEM, meropenem; CAZ, ceftazidime; CIP, ciprofloxacin; PCR, polymerase chain reaction; WGS, whole-genome sequencing; AMR, antimicrobial resistance.
